# Hydroxychloroquine synergizes with the PI3K inhibitor BKM120 to exhibit antitumor efficacy independent of autophagy

**DOI:** 10.1186/s13046-021-02176-2

**Published:** 2021-11-29

**Authors:** Xin Peng, Shaolu Zhang, Wenhui Jiao, Zhenxing Zhong, Yuqi Yang, Francois X. Claret, Moshe Elkabets, Feng Wang, Ran Wang, Yuxu Zhong, Zhe-Sheng Chen, Dexin Kong

**Affiliations:** 1 Tianjin Key Laboratory on Technologies Enabling Development of Clinical Therapeutics and Diagnostics, School of Pharmacy, Tianjin Medical University, Tianjin, 300070 China; 2 Key Laboratory of Immune Microenvironment and Diseases (Ministry of Education), Tianjin Medical University, Tianjin, 300070 China; 3 Department of Systems Biology, The University of Texas MD Anderson Cancer Center, Houston, TX 77030 USA; 4 State Key Laboratory of Toxicology and Medical Countermeasures, Beijing Institute of Pharmacology and Toxicology, Beijing, 100850 China; 5 Department of Pharmaceutical Sciences, College of Pharmacy and Health Sciences, St. John’s University, Queens, NY 11439 USA; 6 The Shraga Segal Department of Microbiology, Immunology and Genetics, Faculty of Health Sciences, Ben-Gurion University of the Negev, 84105 Beer-Sheva, Israel; 7 Department of Genetics, School of Basic Medical Sciences, Tianjin Medical University, Tianjin, 300070 China; 8 School of Medicine, Tianjin Tianshi College, Tianyuan University, Tianjin, 301700 China

**Keywords:** Hydroxychloroquine, BKM120, Autophagy, Homologous recombination repair, ROS, PI3K, NRF2

## Abstract

**Background:**

The critical role of phosphoinositide 3-kinase (PI3K) activation in tumor cell biology has prompted massive efforts to develop PI3K inhibitors (PI3Kis) for cancer therapy. However, recent results from clinical trials have shown only a modest therapeutic efficacy of single-agent PI3Kis in solid tumors. Targeting autophagy has controversial context-dependent effects in cancer treatment. As a FDA-approved lysosomotropic agent, hydroxychloroquine (HCQ) has been well tested as an autophagy inhibitor in preclinical models. Here, we elucidated the novel mechanism of HCQ alone or in combination with PI3Ki BKM120 in the treatment of cancer.

**Methods:**

The antitumor effects of HCQ and BKM120 on three different types of tumor cells were assessed by in vitro PrestoBlue assay, colony formation assay and in vivo zebrafish and nude mouse xenograft models. The involved molecular mechanisms were investigated by MDC staining, LC3 puncta formation assay, immunofluorescent assay, flow cytometric analysis of apoptosis and ROS, qRT-PCR, Western blot, comet assay, homologous recombination (HR) assay and immunohistochemical staining.

**Results:**

HCQ significantly sensitized cancer cells to BKM120 in vitro and in vivo. Interestingly, the sensitization mediated by HCQ could not be phenocopied by treatment with other autophagy inhibitors (Spautin-1, 3-MA and bafilomycin A1) or knockdown of the essential autophagy genes Atg5/Atg7, suggesting that the sensitizing effect might be mediated independent of autophagy status. Mechanistically, HCQ induced ROS production and activated the transcription factor NRF2. In contrast, BKM120 prevented the elimination of ROS by inactivation of NRF2, leading to accumulation of DNA damage. In addition, HCQ activated ATM to enhance HR repair, a high-fidelity repair for DNA double-strand breaks (DSBs) in cells, while BKM120 inhibited HR repair by blocking the phosphorylation of ATM and the expression of BRCA1/2 and Rad51.

**Conclusions:**

Our study revealed that HCQ and BKM120 synergistically increased DSBs in tumor cells and therefore augmented apoptosis, resulting in enhanced antitumor efficacy. Our findings provide a new insight into how HCQ exhibits antitumor efficacy and synergizes with PI3Ki BKM120, and warn that one should consider the “off target” effects of HCQ when used as autophagy inhibitor in the clinical treatment of cancer.

**Supplementary Information:**

The online version contains supplementary material available at 10.1186/s13046-021-02176-2.

## Background

PI3K is known to play vital roles in the regulation of biological functions of tumor cells, such as cell survival, cell growth, and migration [1, 2]. Since the discovery of the first PI3K inhibitor idelalisib (PI3Kp110δ-specific inhibitor) in 2014 [3], there have been 4 PI3K inhibitors approved by the FDA for tumor therapy, including copanlisib (PI3Kp110α and δ-specific inhibitor) [4, 5], duvelisib (PI3Kp110γ and δ-specific inhibitor) [6], and alpelisib (PI3Kp110α-specific inhibitor) [7], and other inhibitors are in clinical trials [8, 9]. New functions of PI3Ks have been discovered in recent years, including their roles in inducing homologous recombination deficiency (HRD) [10] and affecting the tumor microenvironment [11]. However, the antitumor efficacy of PI3Kis alone has been proven limited, and therapeutic resistance continues to be a major impediment to the success of cancer therapy [12, 13]. To circumvent this problem, various combinative therapy strategies of PI3Kis with drugs such as PARP inhibitors, MEK inhibitors and immune checkpoint inhibitors are being tested [14–16]. On the other hand, targeting sole isoforms of PI3K usually causes negative feedback, which may cause therapy resistance [17, 18], providing the rationale for developing pan-PI3K inhibitors such as BKM120 and ZSTK474 [13]. In particular, four phase III clinical trials of BKM120 are ongoing for the treatment of breast and head and neck cancers [19].

Chloroquine (CQ) and its derivative hydroxychloroquine (HCQ), as well-established 4-aminoquinolineanti-malaria drugs [20, 21], have been increasingly used in clinical trials for the treatment of various diseases, such as rheumatoid arthritis, systemic lupus erythematosus [22], and viral infections [23] including COVID-19 [24, 25], due to their extensive pharmacological activities including anti-inflammatory, immunosuppressive and antiviral effects. In addition, they are well-known autophagy inhibitors as lysosomotropic agents [26, 27] and have been tested for enhanced antitumor efficacy in combination with chemotherapeutic drugs. We hypothesized that the combination with autophagy inhibitors might enhance antitumor activity and investigated the antitumor efficacy of BKM120 alone or in combination with the autophagy inhibitor HCQ. As a result, we found that HCQ truly increased the antitumor effect of BKM120 in vitro and in vivo. However, other autophagy inhibitors could not mimic this effect, nor could silence ofAtg5 or Atg7. Moreover, HCQ increased sensitivity to BKM120 even in autophagy-deficient tumor cells, suggesting that such a synergistic effect was independent of autophagy. In this paper, we investigated the mechanism by which HCQ augments the antitumor effect of the PI3Ki BKM120.

## Methods

### Reagents and antibodies

BKM120, HCQ, Spautin-1, 3-MA, bafilomycin A1 (Baf-A1), BAY80-6946, GDC-0941, N-acetylcysteine (NAC) and Z-VAD-FMK (z-VAD) were purchased from Selleck Chemicals (Houston, TX). Tiron (ab146234) was obtained from Abcam. Monodansylcadaverine (MDC) and dichlorodihydrofluorescein diacetate (DCFH-DA) were purchased from Sigma-Aldrich (St. Louis, MO). The antibodies used were as follows: Caspase-3 (Cell Signaling Technology, 9662), Cleaved Caspase-3 (Cell Signaling Technology, 9664), PARP (Cell Signaling Technology, 9532), LC3B (Cell Signaling Technology, 3868), Atg5 (Cell Signaling Technology, 9980), Atg7 (Cell Signaling Technology, 8558), p62 (Cell Signaling Technology, 8025), phospho-Akt (Thr308, Cell Signaling Technology, 13038), phospho-Akt (Ser473, Cell Signaling Technology, 4060), Akt (Cell Signaling Technology, 4685), phospho-PDK1 (Ser241, Cell Signaling Technology, 3438), phospho-mTOR (Ser2448, Cell Signaling Technology, 5536), NRF2 (Cell Signaling Technology, 12712), γH2AX (Abcam, ab81299), BRCA1 (Cell Signaling Technology, 14823, for Western blot), BRCA1 (Abcam, ab16780, for IHC), BRCA2 (Abcam, ab216972), Rad51 (Abcam, 133534), phospho-ATM (S1981, Abcam, ab81292), Ki-67 (Cell Signaling Technology, 9027), β-actin (Cell Signaling Technology, 4970), anti-rabbit IgG, HRP-linked antibody (Cell Signaling Technology, 7074), and Anti-rabbit IgG (H + L), F(ab’)2 Fragment (Alexa Fluor® 488 Conjugate, Cell Signaling Technology, 4412).

### Cell culture

SKOV-3 (ovarian cancer) and DU145 (prostate cancer) cells were purchased from the Cell Bank of the Chinese Academy of Sciences (Shanghai, China), and MKN-1 (gastric cancer) and HBC-5 (breast cancer) cells were graciously provided by the Japanese Foundation for Cancer Research. The cells were cultured in RPMI 1640 medium supplemented with 10% fetal bovine serum (FBS, Gibco), penicillin (100 U/ml), and streptomycin (100 μg/ml) in a humidified incubator with an atmosphere containing 5% CO_2_ at 37 °C. Cells were passaged routinely once every 2–3 days and were maintained for up to a maximum of 20 passages of subculture.

### Cell viability assay

Cells were seeded in 96-well plates at a density of 2 × 10^4^ cells per well in 200 μl of medium. After 24 h, the cells in each well were treated with HCQ or other autophagy inhibitors (Spautin-1, 3-MA, Baf-A1) and were then exposed to BKM120. At each time point, 10 μl of PrestoBlue Cell Viability Reagent (Invitrogen) was added and incubated for the optimized incubation time (1 h) at 37 °C. A Tecan plate reader was used to determine the fluorescence intensity. Cell viability was calculated following normalization to the DMSO vehicle control. Data represent an average of three independent experiments.

### Colony formation assay

Colony formation assay was carried out as described by us previously [28]. Cells were seeded in 24-well plates at a density of 5 × 10^2^ cells per well in 1 ml of medium. After 24 h, cells were treated with the indicated concentrations of HCQ or other autophagy inhibitors (Spautin-1, 3-MA, Baf-A1) in combination with BKM120, BAY80-6946 or GDC-0941 for 72 h and were further incubated in drug-free medium for 7–10 days to form colonies. The colonies were stained with 0.25% crystal violet and 25% methanol in PBS solution for visualization. Colonies with 50 or more cells were counted using ImageJ software with customized parameters that were optimized on the basis of three preliminary manual counts.

### Apoptosis analysis

Apoptosis analysis was carried out as described by us previously [29]. Briefly, cells were treated with HCQ, Spautin-1 or the caspase inhibitor z-VAD and were then exposed to BKM120 for 48 h. After harvesting, the cells were resuspended in 100 μl of binding buffer and incubated with an Annexin V-FITC/PI Apoptosis Detection Kit (BD Biosciences) in the dark for 15 min. Finally, samples were analyzed by flow cytometry in a FACS Verse (BD Biosciences). Data were quantified using FlowJo Software (Tristar).

### Western blot analysis

Western blot analysis was performed as we previously reported [30]. Cells were seeded in 6-well plates. After 24 h, the cells in each well were treated with HCQ or Spautin-1 for 1 h and then BKM120 for 48 h. Total proteins were separated and blotted. The signal was detected by a ChemiDocXRS+ System (BIO-RAD) after exposure to chemiluminescence reagents (BIO-RAD). β-actin served as the loading control.

### Monodansylcadaverine (MDC) staining

MDC staining was used to confirm the existence of autophagic vacuoles as reported previously [31]. Cells were grown on coverslips in 6-well plates. After 24 h, cells were treated with HCQ or Spautin-1 for 1 h and then BKM120 for 48 h. Cells were washed with PBS and incubated with 50 μM MDC at 37 °C for 20 min. The stained cells were washed, fixed with 4% paraformaldehyde for 15 min, and analyzed by fluorescence microscopy (Olympus, BX51) with MetaMorph software. The puncta was quantified using ImageJ.

### Detection of LC3 puncta

LC3 puncta analysis was performed as we previously reported [32]. The GFP-LC3 plasmid or GFP-RFP-LC3 plasmid was transiently transfected into the cells using Lipofectamine 3000 according to the manufacturer’s instructions with slight modification. After incubation overnight, the medium was replaced with fresh growth medium. The cells were incubated with HCQ and then exposed to BKM120 for 48 h. Cells were collected, washed with PBS, and fixed in 4% paraformaldehyde for 30 min at room temperature. Then, the slides were processed and kept in the dark until analysis. Cells were then visualized with Zeiss Confocal microscope LSM880 using 488 nm and 643 nm channels for the presence of GFP-LC3 puncta and RFP-LC3 puncta.

### RNA interference

siRNA knockdown of autophagy-related genes was carried out as reported previously [33]. siRNAs for Atg5, Atg7 and PIK3CA (Atg5: 5′-GGAACAUCACAGUACAUUUTT-3′; Atg7: 5′-GAGACAUGGUCUGAAGAAATT-3′; PIK3CA:5′-CUGAGAAAAUGAAAGCUCACUCUTT-3′) were purchased from Sigma-Aldrich. Cells were transfected with siRNA targeting Atg5, Atg7 or PIK3CA using Lipofectamine 3000 transfection reagent (Invitrogen) according to the manufacturer’s protocol. Non-targeting siRNA (siNT) was used as a negative control. Cells were incubated for an additional 48 h and were then collected for subsequent experiments.

### Immunofluorescence assay

LC3 puncta analysis was performed as we previously reported [34]. The Cells were seeded on coverslips in 24-well plates at a density of 5 × 10^2^ cells per well in 1 ml of medium. After 24 h, cells were cotreated with HCQ and BKM120. After 48 h, cells were fixed with 4% paraformaldehyde and permeabilized with 0.2% Triton X-100 in PBS. Samples were then blocked in 5% donkey serum in the presence of 0.1% Triton X-100 and stained with the primary antibody γH2AX (Abcam, ab81299). After the cells were washed three times with PBS, the secondary antibody coupled to Alexa Fluor 488 was added and incubated for 1 h at room temperature. After being rinsed and washed three times with PBS, slides were mounted using VECTASHIELD mounting medium (Vector Laboratories) containing DAPI. Cells were then visualized with Zeiss Confocal microscope LSM880 for the presence of γH2AX puncta. The puncta was quantified using ImageJ.

### Quantitative RT-PCR

qRT-PCR analysis was performed as we previously reported [35]. Total RNA was extracted with TRIzol (Invitrogen) and synthesized from 1 μg of total RNA using a cDNA Synthesis Kit (BIO-RAD), and RT-PCR was performed with aliquots of cDNA samples mixed with SYBR Green Master Mix (Applied Biosystems). Reactions were carried out in triplicate. The fold difference in transcripts was quantified using the ΔΔC_t_ method. The sequences of the PCR primers were as follows: Nqo1 forward, 5′- CAAATCCTGGAAGGATGGAA-3′; Nqo1 reverse, 5′-GGTTGTCAGTTGGGATGGAC-3′; HMOX1 forward, 5′-CTTCTTCACCTTCCCCAACA-3′; HMOX1 reverse, 5′-GCTCTGGTCCTTGGTGTCAT-3′; 18S rRNA forward, 5′- CAGCCACCCGAGATTGAGCA-3′; 18S rRNA reverse, and 5′-TAGTAGCGACGGGCGGTGTG-3′; 18S rRNA was used as a control to normalize RNA expression.

### Detection of reactive oxygen species (ROS)

The detection of intracellular ROS was carried out as reported by us previously [32]. Briefly, cells were cultured in 6-well plates at a density of 2 × 10^5^ cells per well. After 24 h, HCQ and BKM120 were added. The cells were harvested at the respective time points and incubated with 0.5 μM DCFH-DA in the dark for 30 min. After being washed with PBS, the cell pellets were resuspended in 200 μl of ice-cold PBS for analysis. The fluorescent signal produced was analyzed by a FACS Verse flow cytometer (BD Biosciences). Data were quantified by using FlowJo Software (Tristar).

### Comet assay

The comet assay was carried out as we reported previously [36]. Briefly, Cells were treated with HCQ and BKM120 for 48 h. Cell suspensions were embedded in LM (low melting) Agarose and then solidified at 4 °C. Slides were immersed in lysis solution overnight at 4 °C. Subsequently, the slides were transferred to an electrophoretic box containing 300 mM NaOH and 1 mM Na_2_EDTA (pH > 13) for 30 min at 4 °C before electrophoresis. Thereafter, the slides were rinsed, dehydrated in ethanol, and then stained with SYBR Gold (Invitrogen). Finally, the slides were observed under a laser scanning confocal microscope (FV1000, Olympus). Randomly chosen cells were scored visually by the CASP image-analysis program. % DNA in tails is expressed as the intensity of DNA damage.

### HR repair assay

The HR repair assay was performed as reported previously [37]. Briefly, cells were plated at 2 × 10^5^ cells per well in a 6-well plate. After 24 h, the HR repair reporter substrate direct repeat GFP (DR-GFP) plasmid and the pCBASceI plasmid were transfected into the cells using Lipofectamine 3000 transfection reagent. GFP-expressing plasmid (pEGFP-C1) was used as a transfection efficiency control. Twenty-four hours later, the cells were treated with HCQ and/or BKM120. The cells were harvested after 48 h and resuspended in ice-cold PBS, and the GFP intensity was analyzed using a FACS Verse flow cytometer (BD Biosciences).

### Zebrafish tumor xenograft model

All experimental procedures (including the following nude mouse tumor xenograft model experiments) were approved by the local Laboratory Animal Ethics Committee of the State Key Laboratory of Toxicology and Medical Countermeasures of the Beijing Institute of Pharmacology and Toxicology and performed in accordance with the guidelines for the care and use of laboratory animals. Wild-type AB zebrafish (Daniorerio) were provided by the Academy of Life Sciences of Peking University. Adult zebrafish were maintained under standard laboratory conditions, and embryos were generated by natural pairwise mating. The zebrafish tumor model was established as we reported previously [36]. Two days postfertilization (dpf), the embryos were anesthetized with tricaine (Sigma-Aldrich) and positioned on a Petri dish for microinjections. Tumor cells were incubated with CM-Dil Dye (Invitrogen). A concentration of 10^8^ cells/ml fluorescence-labeled cells was injected into the abdominal perivitelline space of zebrafish embryos. After injection, the tumor-bearing embryos were transferred into a 24-well plate, acclimated at 32 °C for 24 h and then incubated with HCQ and/or BKM120 for 72 h. Tumor growth was imaged using a fluorescence inverted microscope (Olympus). The fluorescence intensity of xenografts was analyzed using ImageJ software. The percentage of tumor surface area of the total larval yolk surface area was calculated to determine the tumor volume.

### Nude mouse tumor xenograft model

The nude mouse tumor xenograft model was established as we reported previously [38]. Four- to five-week-old male BALB/C nude mouse (Vital River Laboratory Animal Technology Company, Beijing) were maintained under specific pathogen-free conditions in the animal facility for 1 week. Suspensions of SKOV-3 and MKN-1 tumor cells (5 ~ 10 × 10^6^ viable cells/mouse) were subcutaneously injected into the right flanks of mice. When reaching 800–1000 mm^3^, the tumor blocks were divided into 2 mm × 2 mm × 2 mm masses and implanted into the right flanks of 30 mice. When the tumors grew to a volume of approximately 100 mm^3^, five mice each were randomly allocated to different groups to be treated orally with vehicle, BKM120, HCQ alone, or the two drugs in combination daily. To evaluate the antitumor effect of the 2 drugs alone or in combination (Combi), tumor volumes were measured every 3 days until the endpoint as (length × width^2^)/2. The body weights of the mice were also measured every 3 days to monitor the possible toxicity. Finally, the mice were euthanized, and tumors from each group were peeled off and weighed for assessment of tumor growth.

### Hematoxylin-eosin (HE), immunohistochemistry and TUNEL assay

HE, TUNEL and immunohistochemical (IHC) staining were carried out as reported by us previously [39]. HE staining was used to detect the pathological changes. Apoptotic cells in tumor tissues were stained with a TUNEL Apoptosis Detection Kit (Beyotime) according to the manufacturer’s protocol. For histological analysis, tumors were fixed overnight in 10% neutral buffered formalin, embedded in paraffin and sectioned at 5-μm thickness using a Leica RM2265 microtome. IHC staining was carried out with an EnVision Detection System HRP. A rabbit/mouse (DAB+) kit (Agilent) was used following the manufacturer’s instructions. Endogenous peroxidase was blocked by incubation with 0.3% hydrogen peroxide for 15 min. Antigen retrieval was performed by boiling the slides in citrate buffer (10 mM, pH 6.0) in a water bath for 20 min. After being rinsed and blocked with 5% bovine serum albumin (BSA), the slides were incubated overnight at 4 °C with primary antibodies, followed by 1 h with labeled Polymer-HRP at room temperature. Subsequently, the slides were exposed to DAB+ Chromogen. Counterstaining with hematoxylin was carried out. After mounting, the slides were observed under an Olympus CX21 microscope, scanned with a high-resolution digital slide scanner (Pannoramic 250, 3DHistech), and quantified by ImageJ software.

### Statistical analysis

Data from three independent experiments are presented and expressed as the mean ± SD. An unpaired, 2-tailed Student’s t test was used for 2-group comparisons. ANOVA with Bonferroni’s correction was used to compare multiple groups. A *p* value of < 0.05 was considered statistically significant. Drug interactions were assessed as CIs, which were calculated using the CalcuSyn software program (Version 2.1, Biosoft). CI < 0.9 represents synergism, 0.9 < CI < 1.1 represents additivity and CI > 1.1 represents antagonism. Before statistical analysis, variations within each group and the assumptions of the tests were checked.

## Results

### HCQ enhanced the antitumor effect of the PI3K inhibitor BKM120

**Fig. 1:**
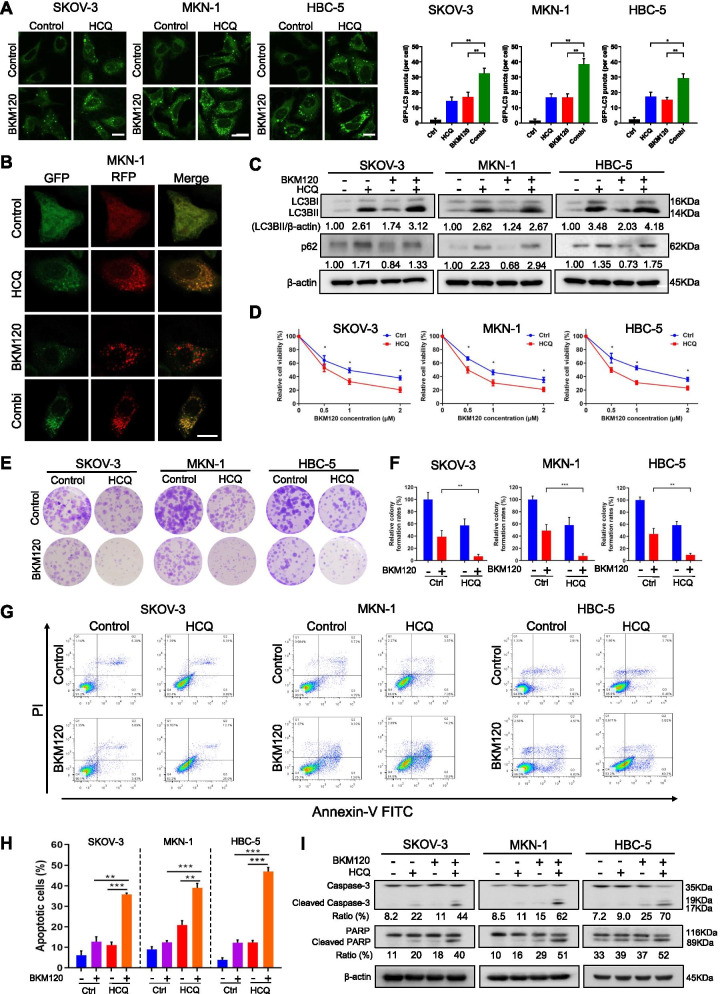
HCQ blocked autophagy and increased the sensitivity of the PI3K inhibitor BKM120 in tumor cells. GFP-LC3 puncta (**A**) and RFP-GFP-LC3 puncta (**B**) in SKOV-3, MKN-1 and HBC-5 tumor cells treated with 1 μM BKM120 or/and 20 μM HCQ (the same concentrations in subsequent studies unless indicated) for 24 h. The number of GFP-LC3 puncta per cell was quantified from 50 randomly selected cells (*n* = 3). **C** Western blot analysis of LC3B and p62 in SKOV-3, MKN-1 and HBC-5 cells treated with BKM120 and/or HCQ for 48 h. **D** Cell viability was measured by PrestoBlue after treatment with BKM120 (0.5 μM, 1 μM and 2 μM) alone or in combination with 20 μM HCQ for 72 h. **E** Colony-forming abilities of cells treated with HCQ and/or BKM120 were determined. Colony quantification is graphed in (**F**). **G** Cells were treated with BKM120 and/or HCQ for 48 h and then subjected to flow cytometric analysis of apoptosis with Annexin V-FITC/PI staining. **H** FACS quantification of the total apoptotic cell population, including Annexin V+/propidium iodide−early apoptotic cells and Annexin V+/propidium iodide+late apoptotic cells. **I** Western blot analysis of Caspase-3 and PARP in SKOV-3, MKN-1 and HBC-5 cells treated with HCQ and/or BKM120. Scale bars, 10 μm. Graphs are normalized to 100% per treatment and are shown as the mean ± SD from three independent experiments; *p < 0.05; **p < 0.01; ***p < 0.001

As a pan-PI3K inhibitor, BKM120 exhibited a favorable antitumor efficacy on various human tumor cells from our JFCR39 drug discovery system [40, 41] regardless of the PIK3CA mutation status. Particularly, apoptosis was induced significantly in SKOV-3, MKN-1 and HBC-5 cells with high caspase 3/7 activity (2-fold to 4-fold higher than other cells in JFCR39 system) [42]. Here, we demonstrated that BKM120 inhibited the proliferation and induced apoptosis and autophagy in SKOV-3, MKN-1 and HBC-5 cells in a dose-dependent manner (Fig. S1A-D), which could be attributed to its inhibitory activity on PI3K pathway (Fig. S1E) [43]. Then, we sought to investigate the effect of HCQ on BKM120-induced autophagy with various assays, including immunofluorescence analysis of GFP-LC3 and GFP-RFP-LC3 puncta, MDC staining, and Western blot analysis of LC3BII (a key autophagosomal surface protein) and p62 (SQSTM1, a key autophagic substrate). Compared with BKM120 treatment alone, combination with HCQ remarkably increased the accumulation of autophagosomes, showing the increased formation of GFP-LC3 puncta (Fig. 1A) and MDC-positive puncta (Fig. S1F, G). We next examined the presence of autophagic flux following BKM120 and HCQ treatment with GFP-RFP-LC3 expressing MKN-1 cells. Increased red vesicles were observed in the cells treated with BKM120, indicative of autophagolysosomes after activation of autophagy. Addition of HCQ increased yellow vesicles (green and red co-localization), suggesting that the BKM120-induced autophagic flux was effectively blocked by HCQ [44] (Fig. 1B). Consistently, the increased expression of LC3BII and p62 suggested that BKM120-induced autophagy was blocked by HCQ at the final stage (Fig. 1C). Notably, combination with HCQ enhanced the anti-proliferative, anti-colony-forming and apoptosis-inducing effects of BKM120 (Fig. 1D-H). Consistent with the annexin V-FITC/PI staining data, the expression levels of cleaved forms of Caspase-3 and PARP detected by Western blot were obviously increased in the combination group (Fig. 1I).

In addition, we used BAY 80-6946 and GDC-0941, to determine whether HCQ could also increase the antitumor effects of other PI3Kis. As shown in the Fig. S2A, the increased expression of LC3BII and p62 suggested that BKM120-induced autophagy was blocked by HCQ at the final stage. HCQ significantly enhanced the anti-proliferative and anti-colony-forming effects of BAY 80-6946 and GDC-0941 (Fig. S2B-D). Consistently, genetic inhibition of PIK3CA with siRNA mimics the effect of BKM120 in MKN-1 cells, with decreased phosphorylation of PI3K downstream factors, such as Akt and mTOR (Fig. S2E). As shown in the Fig. S2F, HCQ could induce more obvious apoptosis in the siPIK3CA group. Overall, HCQ indeed inhibited the autophagy induced by BKM120 while increasing its antitumor effect in vitro.

### Other autophagy inhibitors, such as Spautin-1, could not mimic the effect of HCQ

**Fig. 2:**
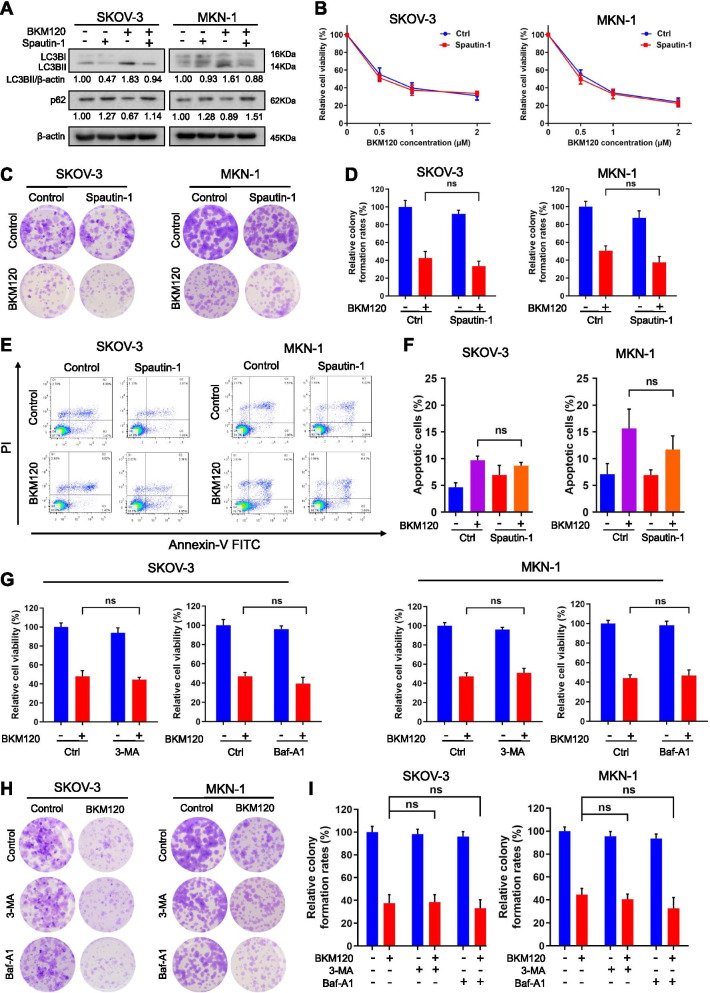
Other autophagy inhibitors did not enhance the anticancer activity of BKM120. **A** Western blot analysis of LC3B and p62 upon treatment with BKM120 and/or 10 μM Spautin-1 for 48 h. **B** Cell viability of SKOV-3 and MKN-1 cells after treatment with various concentrations of BKM120 alone or in combination with 10 μM Spautin-1 for 72 h. **C** The colony formation assay was used to measure the colony-forming abilities of SKOV-3 and MKN-1 cells treated with 10 μM Spautin-1 and/or BKM120. Colony quantification is graphed in (**D**). **E** SKOV-3 and MKN-1 cells were treated with BKM120 and/or 10 μM Spautin-1 for 48 h and subjected to measurement of total apoptotic cells. **F** Quantification of the apoptotic cell population. **G** SKOV-3 and MKN-1cells were treated with BKM120 alone or combined with 3-MA (0.5 mM) or Baf-A1 (1 nM) for 72 h and subjected to measurement of the relative cell viability. **H** Colony-forming abilities of SKOV-3 and MKN-1 cells treated with 3-MA (0.5 mM) or Baf-A1 (1 nM) alone or combined with BKM120. Colony quantification is graphed in (**I**). Graphs are normalized to 100% per treatment and are shown as the mean ± SD from three independent experiments; ns: *p* > 0.05

After demonstrating that HCQ could inhibit autophagy and enhance the cytotoxicity of BKM120, we expected that other autophagy inhibitors could mimic the same effect. First, we determined the effect of Spautin-1, which is known as an early-stage autophagy inhibitor, by increasing proteasomal degradation of class III PI3 kinase complexes [45]. After treatment with Spautin-1, the MDC puncta (Fig. S3A, B) and the expression of LC3BII reduced, and that of p62 increased, suggesting that autophagy was blocked at the early stage [46, 47] (Fig. 2A). Then, we determined the effect of Spautin-1 on the in vitro anticancer activity of BKM120. Interestingly, compared with BKM120 treatment alone, the addition of Spautin-1 did not affect cell viability (Fig. 2B and Fig. S3C), colony-forming ability (Fig. 2C, D and Fig. S3D, E) and cell apoptosis (Fig. 2E, F). In addition, neither 3-MA nor Baf-A1, both of which are recognized autophagy inhibitors, could increase the in vitro anticancer effect of BKM120 (Fig. 2G-I). 3-MA could block autophagy at early stage, while Baf-A1 is a lysosomotropic compound that blocks the autophagic process at late stage, similar to HCQ [48]. Notably, different from the effect of HCQ, treatment with Spautin-1, 3-MA or Baf-A1 alone had no obvious effects on colony formation. Above all, other autophagy inhibitors did not mimic the effect of HCQ to suppress tumor cell growth and to increase the antitumor effect of BKM120, suggesting that HCQ might exhibit the sensitizing effect in a unique way compared with the other autophagy inhibitors.

### Silencing key autophagic genes could not mimic the effect of HCQ

**Fig. 3:**
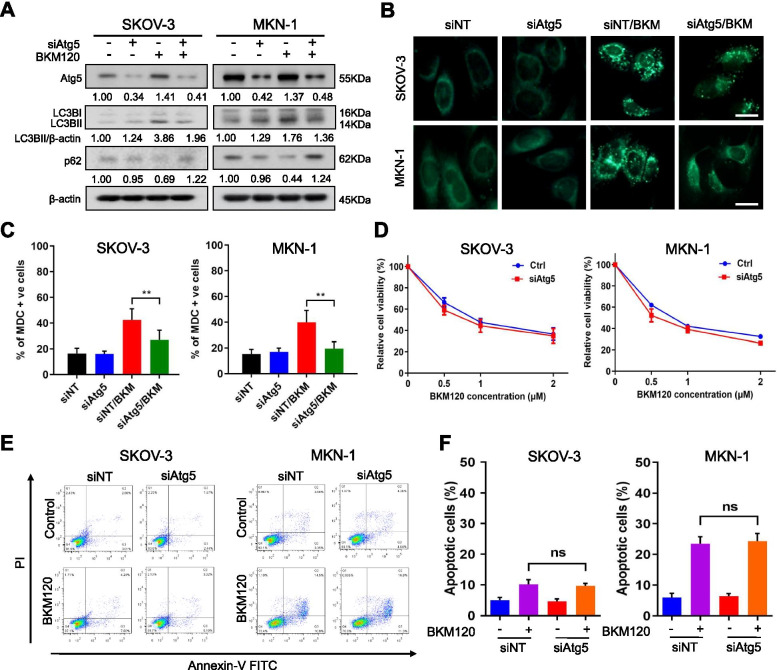
Silencing Atg5 did not affect the anticancer activity of BKM120. **A** Western blot analysis of p62, LC3 and Atg5 in SKOV-3 and MKN-1 cells after transfection with non-targeting siRNA or Atg5 siRNA. SKOV-3 and MKN-1 cells followed by treatment with BKM120 for 48 h. **B**, **C** Representative images of autophagosome puncta in SKOV-3 and MKN-1 cells transfected with non-targeting siRNA or Atg5 siRNA in combination with BKM120 treatment and quantification. Scale bar, 20 μm. **D** SKOV-3 and MKN-1 cells were transfected with or without Atg5 siRNA and were then treated with different concentrations of BKM120 for 72 h to detect cell viability by PrestoBlue assay. **E** Measurement of total apoptotic SKOV-3 and MKN-1 cells after transfection with non-targeting siRNA or Atg5 siRNA in combination with BKM120 treatment. **F** FACS quantification of the total apoptotic cell population. Graphs are normalized to 100% per treatment and are shown as the mean ± SD from three independent experiments; ns: *p* > 0.05; **p < 0.01

To determine whether the antitumor sensitizing effect mediated by HCQ was due to autophagy inhibition, we examined the effect of knocking down the key autophagic gene Atg5 (Fig. S4A). Knocking down Atg5 led to an increase in p62 and decreases in LC3BII in SKOV-3 and MKN-1 cells (Fig. 3A), with an approximately 20% reduction in MDC puncta compared with BKM120 treated groups (Fig. 3B, C), suggesting that autophagy was blocked [46, 47]. However, silencing Atg5 did not affect the effect of BKM120 on cell viability (Fig. 3D) or apoptosis (Fig. 3E, F). We knocked down another key autophagic gene, Atg7 (Fig. S4A), and similar results were obtained (Fig. S4B-D). In addition, silence of the Atg5 or Atg7 also did not sensitize tumor cells to other PI3Kis (Fig. S4E). We conclude that the antitumor effect of BKM120 was not affected by knocking down key autophagic genes, demonstrating that the inhibition of autophagy cannot sensitize tumor cells to BKM120.

### HCQ increased the in vitro anticancer activity of BKM120 in Atg5-deficient cancer cells

**Fig. 4:**
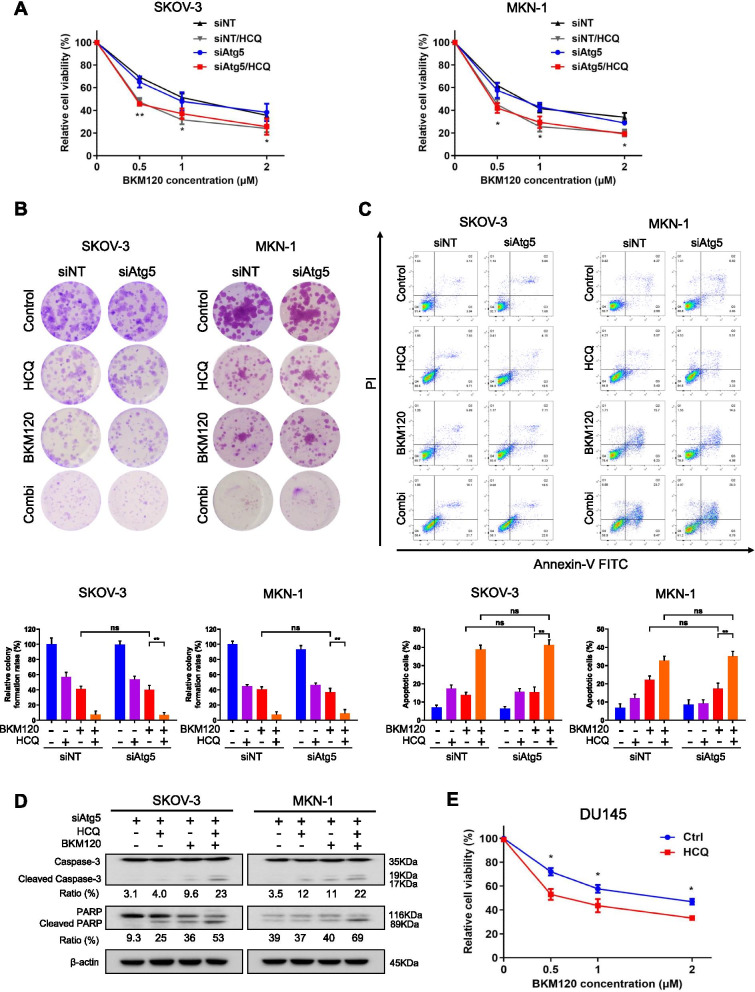
HCQ enhanced the antitumor activity of BKM120 on Atg5-deficient cells. **A** SKOV-3 and MKN-1 cells were transfected with non-targeting or Atg5 siRNA, and then treated with HCQ and various concentrations of BKM120 for 72 h. Cell viability was determined by PrestoBlue assay. **B** SKOV-3 and MKN-1 cells were transfected with or without Atg5 siRNA and were then treated with BKM120 and/or HCQ for 72 h and allowed to recover in fresh media for 7–10 days. The colony-forming ability of the cells was determined. Colony quantification is graphed. **C** SKOV-3 and MKN-1 cells were transfected with or without Atg5 siRNA and then treated with HCQ and/or BKM120 for 48 h. The treated cells were subjected to Annexin V-FITC/PI staining and flow cytometric analysis of apoptosis. The apoptotic cell population is quantified. **D** Western blot analysis of Caspase-3 and PARP in SKOV-3 and MKN-1 cells transfected with Atg5 siRNA and then treated with HCQ and BKM120 for 48 h. **E** DU145 cells were treated with BKM120 alone or in combination with HCQ for 72 h and subjected to cell viability assays. Graphs are normalized to 100% per treatment and shown as the mean ± SD from three independent experiments; ns: *p* > 0.05; *p < 0.05; **p < 0.01; ***p < 0.001

To further demonstrate that the sensitizing effect of HCQ was independent of autophagy status, we investigated the effect of HCQ after knocking down Atg5 in cancer cells. As shown in Fig. 4A, the addition of HCQ significantly increased the antiproliferative activity of BKM120, even in the absence of Atg5. Similarly, knocking down Atg5 did not affect the effect of BKM120 on colony formation, but HCQ significantly enhanced the effect even in Atg5-deficient SKOV-3 and MKN-1 cells, with similar potency to that in parental SKOV-3 and MKN-1 cells (Fig. 4B). Consistently, Atg5 status did not affect apoptosis caused by both drugs (Fig. 4C). The cleaved forms of Caspase-3 and PARP were also significantly increased when cotreated with HCQ and BKM120 in these Atg5-deficient cells (Fig. 4D). In addition, we determined the effects of HCQ and BKM120 on the viability of the prostate cancer DU145 cell line, which is known to be naturally Atg5-deficient [49]. As a result, HCQ showed a sensitizing effect on DU145 cells to BKM120 (Fig. 4E). Overall, we demonstrated that HCQ potentiated the sensitivity of cancer cells to BKM120 independent of autophagy inhibition.

### The combination of HCQ with BKM120 synergistically induced the accumulation of ROS and caspase-dependent apoptosis

**Fig. 5:**
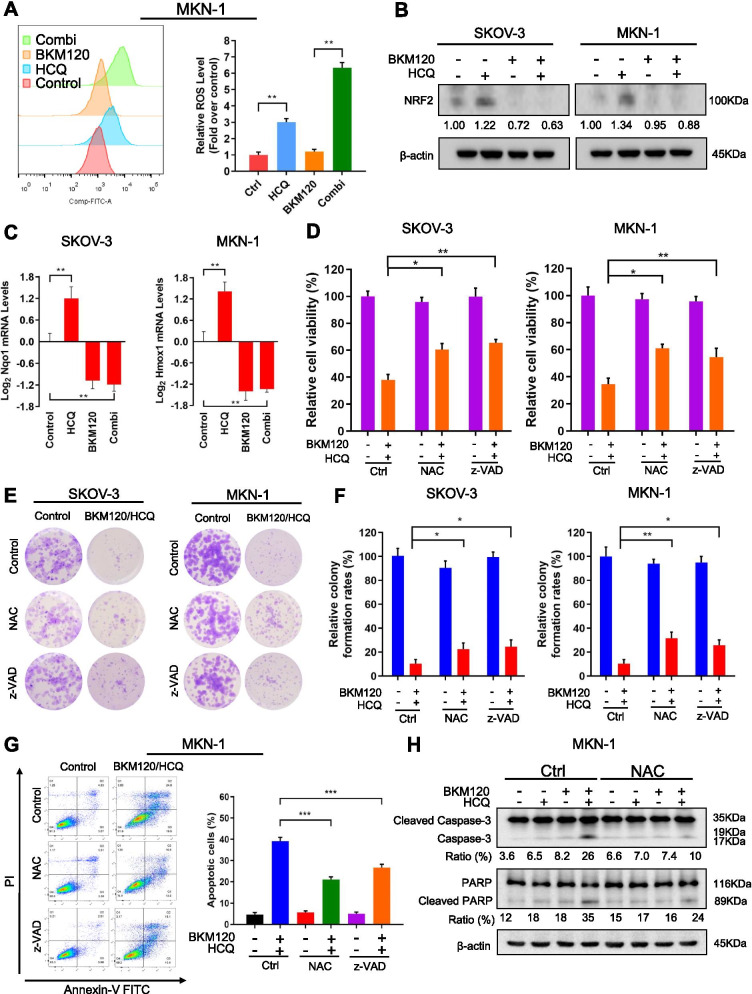
Concurrent treatment with HCQ and BKM120 induced ROS accumulation and apoptosis. **A** Determination of ROS production in MKN-1 cells treated with BKM120 and/or HCQ for 48 h. **B** Western blot analysis of NRF2 in SKOV-3 and MKN-1 cells treated with BKM120 and/or HCQ for 48 h. **C** The mRNA levels of *Nqo1* and *Hmox1* in SKOV-3 cells treated with BKM120 and/or HCQ for 48 h were determined by qRT-PCR. **D** Cell viability of SKOV-3 and MKN-1 cells after treatment with BKM120 and HCQ in combination with NAC or z-VAD for 72 h. **E** Colony-forming ability of SKOV-3 and MKN-1 cells treated with BKM120 and HCQ in the presence or absence of NAC or z-VAD. Colony quantification is graphed in (**F**). **G** SKOV-3 and MKN-1 cells were treated with BKM120 and HCQ in combination with NAC or z-VAD for 48 h and then subjected to Annexin V-FITC/PI staining and flow cytometric analysis of apoptosis. **H** Western blot analysis of Caspase-3 and PARP in SKOV-3 and MKN-1 cells treated with BKM120 or/and HCQ in the presence or absence of NAC for 48 h. Graphs are normalized to 100% per treatment and shown as the mean ± SD from three independent experiments; *p < 0.05; **p < 0.01; ***p < 0.001

Since the sensitizing effect of HCQ could not be attributed to autophagy inhibitory activity, we then tried to delineate the mechanisms behind. The combination of HCQ with BKM120 led to a strong synergetic antiproliferative effect on both SKOV-3 and MKN-1 cells, with CI values far less than 1 (Fig. S5A,B). HCQ alone induced apoptosis in a concentration-dependent manner, with an increase in cleaved PARP (Fig. S4C-E). Furthermore, treatment with HCQ time-dependently increased the production of ROS (Fig. S4F, G). Interestingly, it is worth noting that BKM120 alone did not induce significant ROS production but led to a high increase in ROS accumulation when combined with HCQ (Fig. 5A). Silencing Atg5 in combination with BKM120 did not mimic the effect of HCQ combined with BKM120 on ROS production (Fig. S5H, I). Moreover, HCQ alone increased the expression of NRF2,a transcription factor encoded by the NFE2L2 gene,which mediates the transcription of antioxidative genes [50]. In contrast, BKM120 alone or in combination with HCQ inhibited the expression of NRF2 (Fig. 5B). We further examined their effect on the mRNA expression of NRF2-targeted genes such as*Nqo1* and *Hmox1*. Consistently, HCQ increased the expression of *Nqo1* and *Hmox1*, while BKM120 potently inhibited them (Fig. 5C). Previous reports showed that the excessiveaccumulation of ROS in cells may lead to caspase-dependent cell death [51, 52]. We found that pretreatment with the antioxidant NAC or the pancaspase inhibitor z-VAD could partly rescued the cytotoxic effects of HCQ and BKM120, showing reduced effects on the inhibition of cell viability (Fig. 5D) and cell colony formation (Fig. 5E, F), as well as the induction of apoptosis (Fig. 5G). The cleaved forms of Caspase-3 and PARP were also reduced in the presence of NAC (Fig. 5H). However, the rescue effects were limited, indicating other mechanisms may exist to mediate the tumor cell death. In addition, in order to further confirm that ROS induction is involved in the mechanism of HCQ to enhance the antitumor activity of BKM120, two recognized ROS inducers, doxorubicin and cisplatin [53, 54], were used to combine with BKM120. As shown in Fig. S6A and B, addition of either doxorubicin or cisplatin significantly enhanced ROS accumulation and cell death in MKN-1 cells, compared with treatment by BKM120 alone. The above results suggested that the synergistic antitumor effect of the combination of HCQ and BKM120 was mediated at least partially through the accumulation of ROS and the induction of caspase-dependent apoptosis.

### HCQ and BKM120 synergistically induced DSBs with different roles in HR repair

**Fig. 6:**
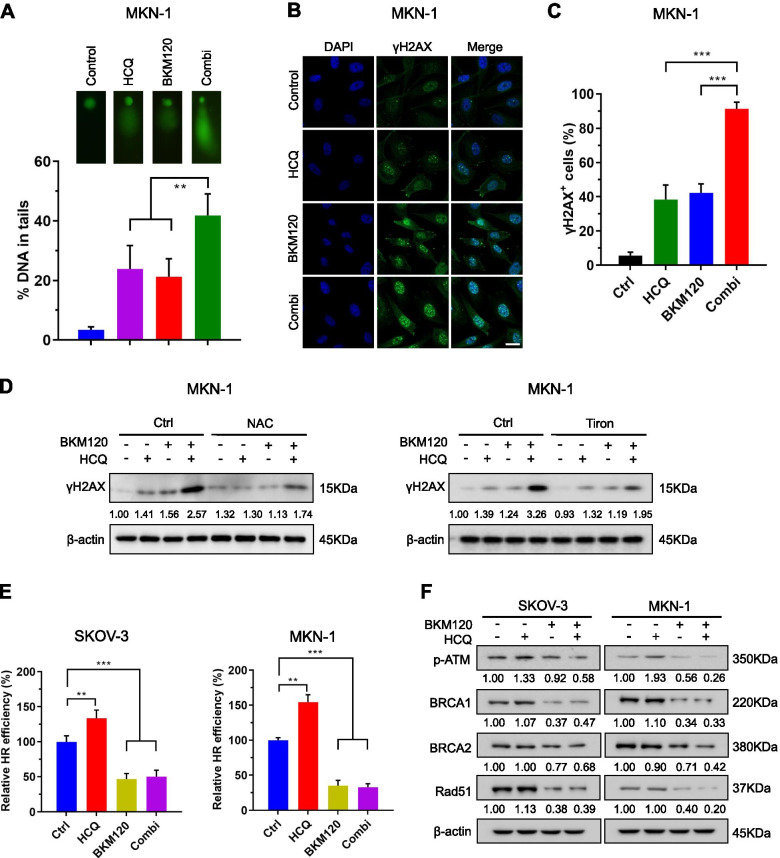
HCQ and BKM120 cooperated to induce DSBs. **A** Comet assay of MKN-1 cells treated with HCQ and/or BKM120 for 48 h. The %DNA in tails was quantified to represent DNA damage intensity. **B** Representative images of immunofluorescence staining of γH2AX in MKN-1 cells treated with HCQ and/or BKM120 for 48 h. Cell nuclei were stained with DAPI. Scale bar, 20 μm. The percentage of γH2AX foci positive cells (> 5 foci per cell) is shown in (**C**). **D** Western blot analysis of γH2AX in MKN-1 cells treated with BKM120 and/or HCQ in the presence or absence of NAC or Tiron for 48 h. **E** HR reporter assay was used to detect the effect of the drugs on HR repair efficiency in SKOV-3 and MKN-1 cells. **F** Western blot analysis of p-ATM, BRCA1, BRCA2 and Rad51 in SKOV-3 and MKN-1 cells treated with each drug alone or in combination for 48 h. Graphs are normalized to the untreated group and shown as the mean ± SD from three independent experiments; **p < 0.01; ***p < 0.001

ROS accumulation is a major cause of DNA damage, of which double-strand breaks (DSBs) are fatal [55, 56]. The comet assay indicated that the combination of HCQ and BKM120 led to increased DNA fragmentation, suggesting more severe DNA damage (Fig. 6A). Immunofluorescence staining and Western blot results showed that the foci formation and abundance of γH2AX increased, indicating augmented DSBs (Fig. 6B, C). However, pretreatment with NAC or Tiron, both of which are ROS scavengers, led to a reduction of γH2AX in combination group, suggesting that ROS accumulation at least partially contributed to the DSB caused by the combined administration of HCQ and BKM120 (Fig. 6D and Fig. S7A). We also used an HR reporter assay to investigate the effect on HR repair efficiency [37]. As shown in Fig. 6E and Fig. S7B, HCQ enhanced HR repair efficiency, while BKM120 dramatically inhibited it in cancer cells. We next sought to gain insight into how HR repair was affected by both drugs. Western blot analysis showed that HCQ increased the phosphorylation of ATM (Fig. 6F), an essential regulator in the HR repair process, by transducing HR repair signals to downstream effectors by phosphorylating critical protein substrates, including BRCA1/2 [57]. In contrast, the phosphorylation of ATM was inhibited by BKM120, as well as the expression of the key downstream HR molecules BRCA1, BRCA2 and Rad51 (Fig. 6F). In summary, HCQ and BKM120 synergistically caused DSBs in cancer cells, with different roles in HR repair.

### In vivo synergetic effect of HCQ and BKM120

**Fig. 7:**
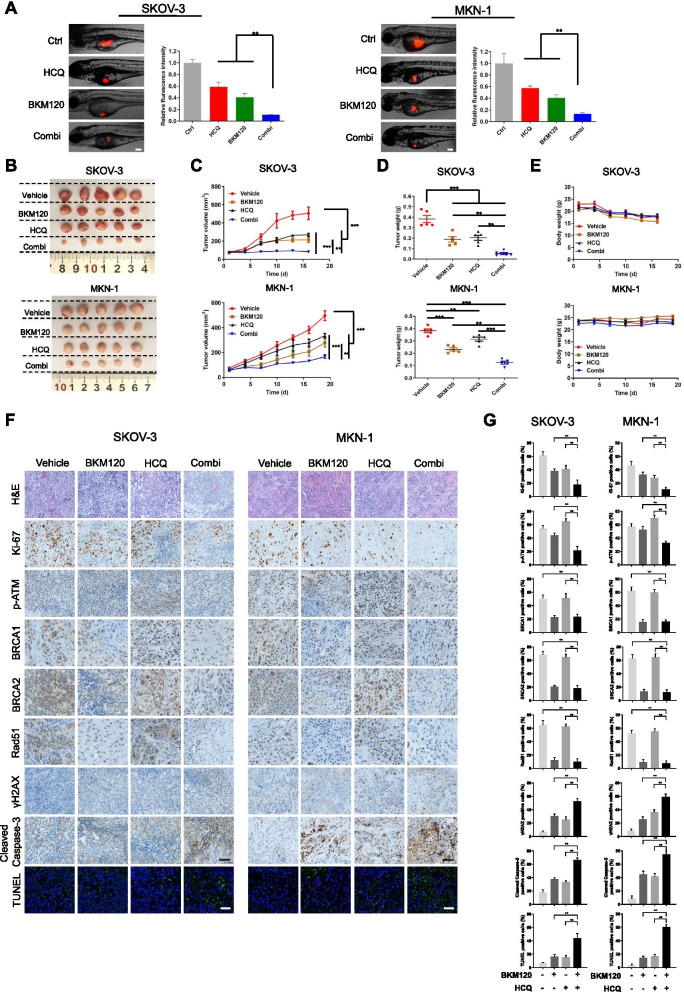
BKM120/HCQ combination treatment inhibited tumor growth in zebrafish and nude mouse xenograft models. **A** SKOV-3 and MKN-1 cells were labeled with Dil (red fluorescence) and injected into each embryo. The injected embryos were transferred to a 24-well plate containing 20 μM HCQ and/or 2 μM BKM120 and incubated for 72 h. The embryos were imaged with a fluorescence microscope to detect tumor growth and the resulting data were quantified, *n* = 8 in each group. Scale bar, 50 μm. **B** Representative images of the tumors from each group of nude mice upon treatment with vehicle, BKM120 (20 mg/kg/day), HCQ (30 mg/kg/day), or both drugs for the respective time periods. **C** Relative tumor volume at various time points of each group of nude mice (normalized to day 0). **D** Measurement of tumor weight after treatment for the respective time periods. **E** Time-course measurements of mouse weights every 3 days. **F** Representative images of H&E and TUNEL staining, and immunohistochemical staining analyses of Ki-67, p-ATM, BRCA1, BRCA2, Rad51, γH2AX and cleaved Caspase-3 in tumor tissues. Apoptotic cells in tumor tissues were detected by TUNEL staining. **G** Statistical quantification of (**F**). Scale bars, 50 μm. Graphs are normalized to the untreated group and shown as the mean ± SD from three independent experiments; **p < 0.01; ***p < 0.001

After demonstrating the in vitro synergetic effect of HCQ and BKM120, we investigated the in vivo antitumor efficacy of the two drugs using SKOV-3 and MKN-1 zebrafish and nude mouse xenograft models. As shown in Fig. 7A, the fluorescence intensity, which indicated the tumor size in zebrafish models, was significantly reduced in the HCQ, BKM120 and the combination groups after treatment for 3 days. Furthermore, compared with either drug alone, the combination treatment resulted in a significantly reduction in tumor growth. For antitumor efficacy in nude mouse xenograft models, both HCQ and BKM120 significantly inhibited SKOV-3 and MKN-1 tumor growth, while the combination further enhanced the efficacy compared with either drug alone (Fig. 7B-D). There was no apparent change in body weight indicated between the combination and either the HCQ or BKM120 groups, suggesting no major toxicity caused by the combination treatment (Fig. 7E). Next, immunohistochemical and TUNEL staining assays were carried out with the tumor tissues. As indicated in Fig. 7F, SKOV-3 and MKN-1 cells showed obvious atypia of different sizes, various shapes, and patch or cord arrangements. The combination treatment reduced Ki-67 levels and increased cleaved Caspase-3 expression in TUNEL-positive cells, suggesting the enhancement of antiproliferative and apoptosis-inducing effects. The HR-related proteins including p-ATM, BRCA1/2, RAD51 and γH2AX were also showed changes in consistent with the in vitro results (Fig. 7G). Above all, the combination of HCQ and BKM120 resulted in significantly enhanced in vivo antitumor efficacy, which might be attributed to their synergetic activity on the accumulation of ROS and DSBs.

**Fig. 8:**
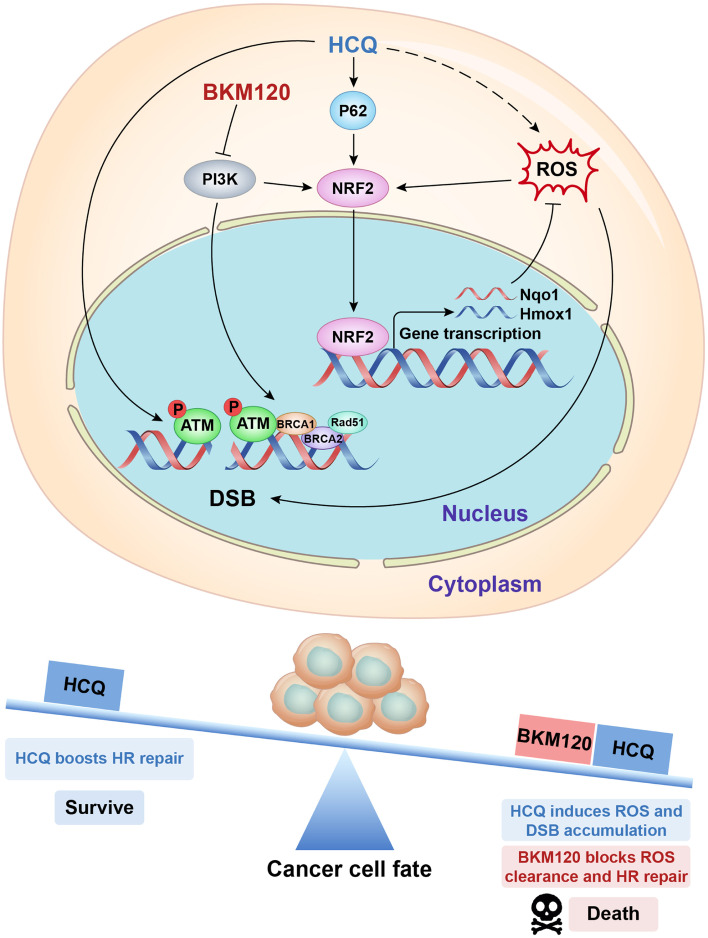
Schematic diagram of the rationale of the combination of HCQ with BKM120 for cancer therapy

Taken together, our data indicated that HCQ synergized with BKM120 to exhibit enhanced antitumor activity independent of autophagy. Mechanistically, HCQ increased ROS production, and BKM120 blocked ROS clearance by downregulating NRF2. Increased ROS accumulation in cancer cells serves as the major source to induce DSBs. Simultaneously, HCQ activated ATM to enhance HR repair efficiency, which in turn ameliorated DSBs. Nevertheless, the HR repair process was significantly impaired by BKM120, resulting in the accumulation of unrepaired exogenous and endogenous DSBs and subsequent cell apoptosis (Fig. 8).

## Discussion

CQ and its derivative HCQ were originally developed as antimalarial drugs [58] and were later repurposed for the treatment of many diseases due to their broad pharmacological effects [59–61]. In cancer clinical trials, HCQ and CQ have been focused on as autophagy inhibitors to sensitize tumor cells to anticancer agents. Previous reports have shown that HCQ and CQ can enhance the anticancer effects of the Akt inhibitor AZD5368 and the CDK4/6 inhibitor palbociclib through blockade of autophagy [62, 63]. We confirmed that HCQ blocked BKM120-induced autophagy and sensitized SKOV-3, MKN-1 and HBC-5 tumor cells to BKM120. Unexpectedly, we couldn’t recapitulate the sensitizing effect of HCQ with other autophagy inhibitors, suggesting that HCQ might have a unique mode of action. Next, we sought to investigate the effect of genetic inhibition of autophagy with siRNA on the antitumor activity of BKM120. Interestingly, knockdown of the essential autophagy factors Atg5 and Atg7 did not affect the antitumor potency of BKM120. Moreover, HCQ could still enhance the antitumor activity of BKM120 in the absence of Atg5. We therefore hypothesized that autophagy might play a dispensable role in HCQ’s sensitizing effect. In other words, in addition to the inhibition of autophagy, HCQ might exhibit an “off-target” effect on tumor cells. There have been fragmentary reports that CQ or HCQ may have unique anticancer mechanisms in addition to blocking autophagy. Maycotte et al. [64] found that CQ enhanced chemotherapeutic efficiency independently of autophagy in mouse breast cancer cells. Maes et al. [65] proposed a rationale for restraining cancer metastasis to improve chemotherapy through autophagy-independent vessel normalization using CQ. Piao et al. [26] claimed that the expression of canonical autophagy genes was not associated with the sensitivity of HCQ. In KRAS-driven cancers, Atg7 depletion did not affect the antiproliferative effect of CQ, suggesting that the antitumor effect of CQ might be independent of autophagy [66].

Given that our findings showed that HCQ synergized with BKM120 to exhibit antitumor effects independently of autophagy, we next sought to gain mechanistic insight into how the synergistic effect occurred. As a lysosomotropic agent, HCQ accumulates in the lysosome, impairing the elimination of damaged mitochondria, which are known as the source of ROS [67]. Interestingly, we also demonstrated that autophagy was not necessarily associated with the increase in ROS production by HCQ, as inhibition of autophagy alone or in combination with BKM120 did not lead to ROS accumulation. We then noticed that HCQ alone could induce cancer cell apoptosis at a relatively higher concentration (> 40 μM) than that used in the combination treatment. HCQ also promoted ROS production in a time-dependent manner. In parallel, HCQ increased NRF2 protein abundance, which partially antagonized the accumulation of ROS. The upregulation of NRF2 could be explained by the competitive binding of p62 to NRF2 with KEAP1, therefore leading to NRF2 stabilization and promoting the transcription of NRF2 target genes [68], such as *Nqo1* and *Hmox1*. The KEAP1-NRF2 pathway is known to play a vital role in the elimination of ROS [69–71]. Under normal conditions, NRF2 is degraded in the cytoplasm by interacting with KEAP1 as a substrate of the ubiquitination system. However, excessive production of ROS directly modifies cysteine residues in KEAP1, leading to NRF2 stabilization and translocation into the nucleus [72]. In addition, the PI3K pathway was reported to increase the stability and the transcriptional activity of NRF2 and therefore upregulate the target genes of NRF2 [73–75]. As a pan-PI3K inhibitor, BKM120 effectively blocked the PI3K pathway and inhibited NRF2 expression. Even when BKM120 was combined with HCQ, the expressions of NRF2 and its antioxidant target genes such as *Nqo1* and *Hmox1* still decreased to a similar extent to those after BKM120 treatment alone, indicating inhibition of PI3K by BKM120 may be sufficient to reverse the activation of NRF2 caused by HCQ, and thus aggravating ROS accumulation.

The cytotoxicity of ROS is known to be mainly dependent on damage to DNA, especially DSBs [55, 56]. Consistently, we demonstrated that the combination of HCQ and BKM120 induced DSBs, which was at least partially due to the increase in ROS production. Interestingly, the two drugs not only played different roles in promoting ROS production and DSB accumulation but also affected DNA damage repair in different ways. Approximately two decades ago, CQ was reported to change chromatin structure by inserting DNA fragments and subsequently activating the HR repair protein ATM [76, 77]. Subsequently, CQ or HCQ was often used as an ATM activator [78]. In recent years, CQ has also been demonstrated to activate ATM to enhance HR repair efficiency [79]. Two groups reported that PI3K inhibition can result in the failure of HR repair by the downregulation of BRCA1/2 [16, 80]. Downstream factors of PI3K, such as Akt and mTOR, have also been found to participate in the HR repair process [81, 82]. Based on these findings, a number of clinical studies are being carried out on PI3Kis in combination with RARP inhibitors in various types of cancers [83, 84]. In this study, HR reporter assay results indicated that HCQ alone improved HR repair efficiency. BKM120 alone or in combination with HCQ induced HR deficiency (HRD) in SKOV-3, MKN1 and HBC-5 cancer cells, which are HR-proficient cell lines, as indicated in our data and previous reports [85, 86]. Furthermore, HCQ enhanced the phosphorylation of ATM, the active form of ATM, while BKM120 inhibited this process, consistent with previous reports [87, 88] that suppression of PI3K could block the phosphorylation of ATM. Moreover, BKM120 inhibited the expression of BRCA1, BRCA2 and Rad51, the key HR repair molecules downstream of ATM. Therefore, the dual inhibition of ATM and its key downstream factors allowed BKM120 to completely reverse the HCQ-improved HR repair efficiency in the tumor cells. Unrepaired DSBs eventually led to cell apoptosis [89], as demonstrated in our results.

## Conclusions

In summary, our study demonstrated that HCQ combined with BKM120 could exhibit synergistic effects on SKOV-3, MKN-1 and HBC-5 tumor cells by manipulating the ROS clearance and HR repair processes independent of autophagy, suggesting a promising combination strategy of CQ or HCQ together with PI3Kis for cancer therapy. In addition, we elucidated the self-contradictory roles of HCQ in ROS production and DNA damage accumulation, providing new insight into the antitumor effect of HCQ. Notably, the “off-target” effects of HCQ should be considered when used as an autophagy inhibitor in the clinical treatment of cancer.

## Supplementary Information

**Additional file 1: Figure S1.** Inhibition of PI3K suppressed cell proliferation and induced apoptosis in tumor cells. (A) The three tumor cell lines were treated with different concentrations of BKM120 for 48 h. Cell viability was determined by PrestoBlue assay. (B) The cells were treated with indicated concentrations of BKM120 for 48 h and subjected to Annexin V-FITC/PI staining and flow cytometric analysis of apoptosis. (C) FACS quantification of total apoptotic cell population including Annexin V+/propidium iodide−early apoptotic cells and Annexin V+/propidium iodide+late apoptotic cells. (D) Western blot analysis of Caspase-3, PARP, LC3B and p62 after treated with BKM120 for 48 h. (E) Western blot analysis of p-Akt, Akt and p-mTOR after treatment with BKM120 for 48 h. (F) Representative images of autophagosome puncta in three cell lines treated with BKM120 or/and HCQ for 24 h. Scale bars, 20 μm. (G) Quantification of autophagosome puncta positive cells (> 10 puncta per cell). All data are mean ± SD from three independent experiments; *p < 0.05; **p < 0.01; ***p < 0.001.

**Additional file 2: Figure S2.** HCQ increased the sensitivity of the other PI3K inhibitors in MKN-1 cells. (A) Western blot analysis of LC3B and p62 in MKN-1 cells treated with BKM120 and/or HCQ for 48 h. (B) Cell viability was measured by PrestoBlue after treatment with BAY 80-6946/GDC-0941 alone or in combination with 20 μM HCQ for 72 h. (C) Colony-forming abilities of cells treated with HCQ and/or BAY 80-6946/GDC-0941 were determined. Colony quantification is graphed in (D). (E) Western blot analysis of PI3K-p110α, p-Akt, Akt and p-mTOR after siRNA knockdown of PI3KCA. (F) The cells were treated with HCQ for 48 h in the presence or absence of PI3KCA siRNA, and cell apoptosis was measured. Graphs are normalized to 100% per treatment and shown as mean ± SD from three independent experiments; *p < 0.05; **p < 0.01.

**Additional file 3: Figure S3.** The effect of Spautin-1 on the autophagy and cytotoxity caused by BKM120. Representative images of autophagosome puncta (A) in SKOV-3 and MKN-1 treated with BKM120 (1 μM), Spautin-1 (10 μM), alone or in combination for 24 h, and the quantification (B). Scale bars, 20 μm. (C) Cell viability of HBC-5 cells after treated with different concentrations of BKM120 alone or in combination with 10 μM Spautin-1 for 72 h. (D) Colony forming ability of HBC-5 cells after treated with Spautin-1 or/and BKM120. Graph of colony quantification is shown in (E). Graphs are normalized to 100% per treatment and shown as mean ± SD from three independent experiments; ns: *p* > 0.05; **p < 0.01.

**Additional file 4: Figure S4.** Silence of Atg5 or Atg7 did not affect the anticancer activity of BKM120 and other PI3K inhibitors. (A) Western blot analysis of Atg5 and Atg7 in SKOV-3 and MKN-1 cells after transfection with non-targeting siRNA (siNT), Atg5 and Atg7 siRNA, respectively. (B) Cell viability of SKOV-3 and MKN-1 cells after transfected with Atg7 siRNA, followed by treatment of BKM120 for 72 h. (C) Colony forming ability of SKOV-3 and MKN-1 cells after transfected with non-target siRNA or Atg7 siRNA and treated with BKM120 for 72 h and allowed to recover in fresh media for 7–10 days. Graph of colony quantification is shown in (D). (E) Cell viability of MKN-1 cells after transfected with Atg5 or Atg7 siRNA, followed by treatment of BAY 80-6946 or GDC-0941 for 72 h. Graphs are normalized to 100% per treatment and shown as mean ± SD from three independent experiments; ns: *p* > 0.05.

**Additional file 5: Figure S5.** The effect of HCQ on ROS production and the synergetic effect with BKM120 on proliferation inhibition in SKOV-3 and MKN-1 cells. (A) Cell viability of SKOV-3 and MKN-1 cells after treatment with BKM120 or HCQ alone or in combination for 72 h. (B) The drug combination was analyzed using CalcuSyn software and the resulting CI-Fa plots are shown. (C) SKOV-3 and MKN-1 cells were treated with various concentrations of HCQ for 48 h and then subjected to apoptosis analysis. (D) FACS quantification of total apoptotic cell population. (E) Western blot analysis of PARP in SKOV-3 and MKN-1 cells treated with HCQ for 48 h. ROS levels (F) in SKOV-3 and MKN-1 cells were determined after treatment with 40 μM HCQ for 6 and 48 h, respectively, which were quantified as in (G). ROS level (H) in MKN-1 cells after transfected with Atg5 siRNA alone or in combination with BKM120 for 48 h was determined, and quantified as in (I). (J) Quantification of NRF2 expression after correction with the β-actin control. Graphs are shown as mean ± SD from three independent experiments; ns: *p* > 0.05; *p < 0.05; **p < 0.01.

**Additional file 6: Figure S6.** ROS inducers synergized with BKM120 to exhibit anti-proliferative effect in MKN-1 cells. (A) Determination of ROS production in MKN-1 cells treated with BKM120 and/or Cisplatin/Doxorubicin for 48 h. (B) Cell viability was measured by PrestoBlue after treatment with BKM120 alone or in combination with Cisplatin/Doxorubicin for 72 h. Graphs are normalized to the untreated group and shown as the mean ± SD from three independent experiments; *p < 0.05; **p < 0.01; ***p < 0.001.

**Additional file 7: Figure S7.** The effect of combination of HCQ and BKM120 on DSBs in tumor cells. (A) Quantification of γH2AX levels after correction with the β-actin control. (B) The HR reporter assay was used to detect the effect of the drugs on HR repair efficiency in HBC-5 cells. (C) Quantification of p-ATM levels after correction with the β-actin control; Graphs are shown as mean ± SD from three independent experiments; *p < 0.05; **p < 0.01.

## Data Availability

The datasets used and/or analyzed during the current study are available from the corresponding author on reasonable request.

## Abbreviations

Baf-A1: Bafilomycin A1
BSA: Bovine serum albumin
CQ: Chloroquine
DCFH-DA: Dichloro-dihydro-fluorescein diacetate
dpf: Days postfertilization
DSB: DNA double-strand break
HCQ: Hydroxychloroquine
HE: Hematoxylin-eosin
HRD: Homologous recombination deficient
IHC: Immunohistochemistry
MDC: Monodansylcadaverine
PI3Ki: PI3K inhibitor
ROS: Reactive oxygen species
siNT: Non-targeting siRNA
z-VAD: Z-VAD-FMK
