# Correction to: Hydroxychloroquine synergizes with the PI3K inhibitor BKM120 to exhibit antitumor efficacy independent of autophagy

**DOI:** 10.1186/s13046-021-02218-9

**Published:** 2021-12-20

**Authors:** Xin Peng, Shaolu Zhang, Wenhui Jiao, Zhenxing Zhong, Yuqi Yang, Francois X. Claret, Moshe Elkabets, Feng Wang, Ran Wang, Yuxu Zhong, Zhe-Sheng Chen, Dexin Kong

**Affiliations:** 1 Tianjin Key Laboratory on Technologies Enabling Development of Clinical Therapeutics and Diagnostics, School of Pharmacy, Tianjin Medical University, Tianjin, 300070 China; 2 Key Laboratory of Immune Microenvironment and Diseases (Ministry of Education), Tianjin Medical University, Tianjin, 300070 China; 3 Department of Systems Biology, The University of Texas MD Anderson Cancer Center, Houston, TX 77030 USA; 4 State Key Laboratory of Toxicology and Medical Countermeasures, Beijing Institute of Pharmacology and Toxicology, Beijing, 100850 China; 5 Department of Pharmaceutical Sciences, College of Pharmacy and Health Sciences, St. John’s University, Queens, New York, NY 11439 USA; 6 The Shraga Segal Department of Microbiology, Immunology and Genetics, Faculty of Health Sciences, Ben-Gurion University of the Negev, 84105 Beer-Sheva, Israel; 7 Department of Genetics, School of Basic Medical Sciences, Tianjin Medical University, Tianjin, 300070 China; 8 School of Medicine, Tianjin Tianshi College, Tianyuan University, Tianjin, 301700 China

**Correction to: J Exp Clin Cancer Res 40, 374 (2021)**

**https://doi.org/10.1186/s13046-021-02176-2**

Following publication of the original article [1], author found an error about the information of the last author, Dexin Kong, which was made in the production stage of the publication. The information indicating Dexin Kong as a corresponding author was missing, including the symbol "*" on his name and his email address. Such information has been added.

The correction does not have any effect on the results or conclusions of the paper. The original article has been corrected.
