# Supplementary figures and images for: Hydroxychloroquine synergizes with the PI3K inhibitor BKM120 to exhibit antitumor efficacy independent of autophagy
Source: J Exp Clin Cancer Res. 2021 Nov 29;40:374. doi: 10.1186/s13046-021-02176-2 (PMC8628289; doi:10.1186/s13046-021-02176-2)

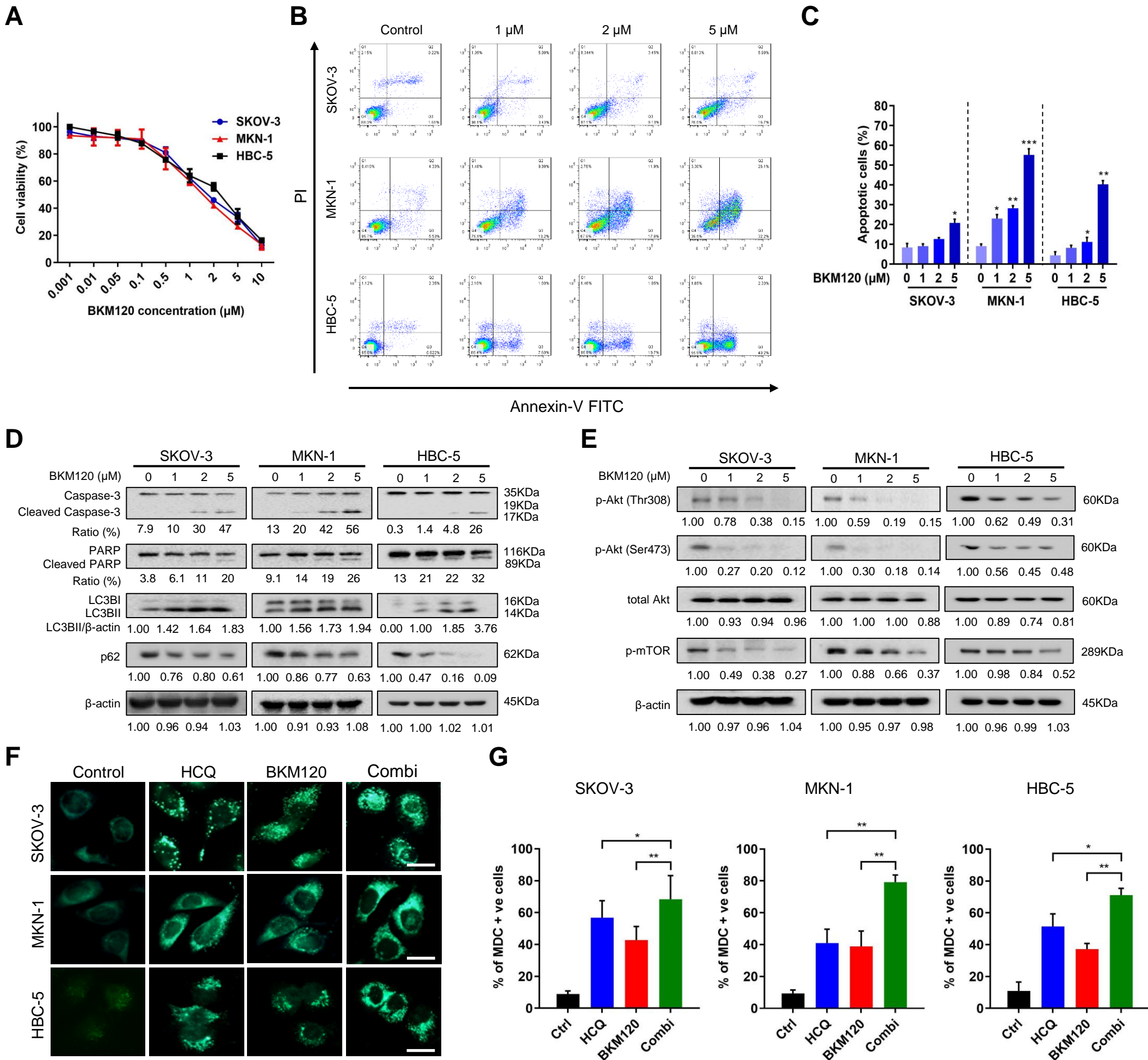

Supplement: Supplementary file 1 — Additional file 1: Figure S1. Inhibition of PI3K suppressed cell proliferation and induced apoptosis in tumor cells. (A) The three tumor cell lines were treated with different concentrations of BKM120 for 48 h. Cell viability was determined by PrestoBlue assay. (B) The cells were treated with indicated concentrations of BKM120 for 48 h and subjected to Annexin V-FITC/PI staining and flow cytometric analysis of apoptosis. (C) FACS quantification of total apoptotic cell population including Annexin V+/propidium iodide−early apoptotic cells and Annexin V+/propidium iodide+late apoptotic cells. (D) Western blot analysis of Caspase-3, PARP, LC3B and p62 after treated with BKM120 for 48 h. (E) Western blot analysis of p-Akt, Akt and p-mTOR after treatment with BKM120 for 48 h. (F) Representative images of autophagosome puncta in three cell lines treated with BKM120 or/and HCQ for 24 h. Scale bars, 20 μm. (G) Quantification of autophagosome puncta positive cells (> 10 puncta per cell). All data are mean ± SD from three independent experiments; *p < 0.05; **p < 0.01; ***p < 0.001. [file 13046_2021_2176_MOESM1_ESM.pdf]

A

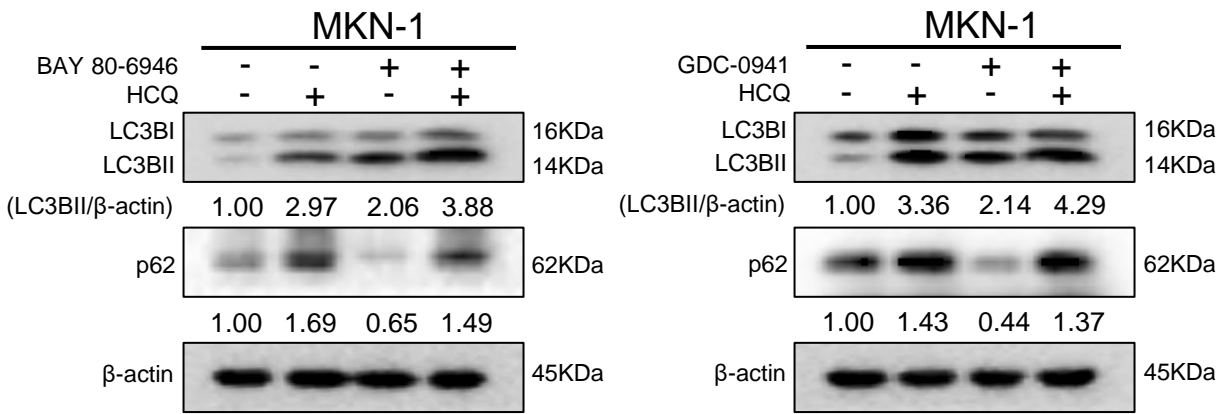

B

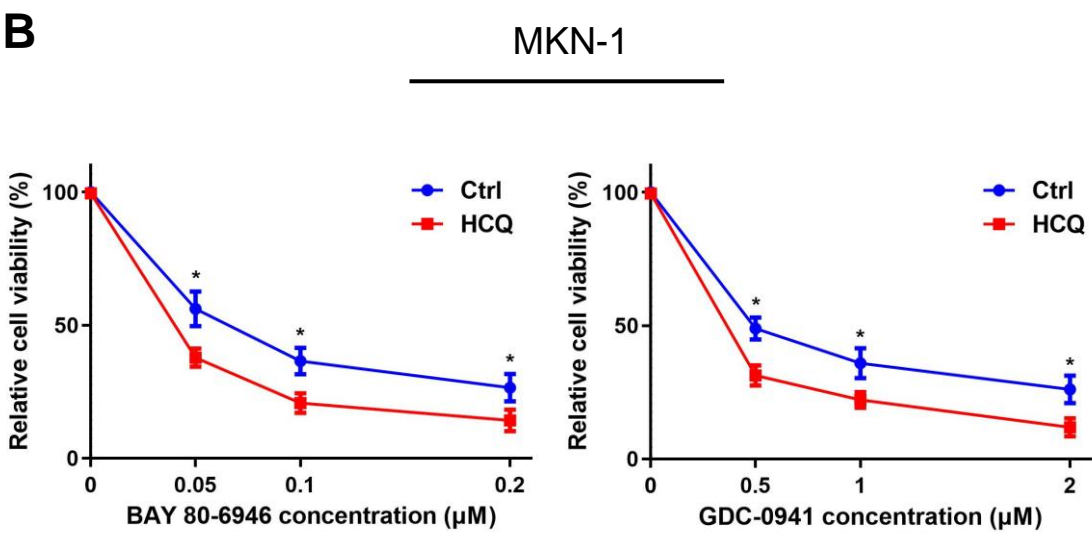

C

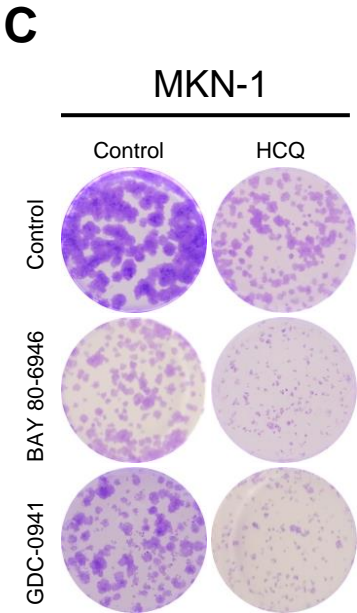

D

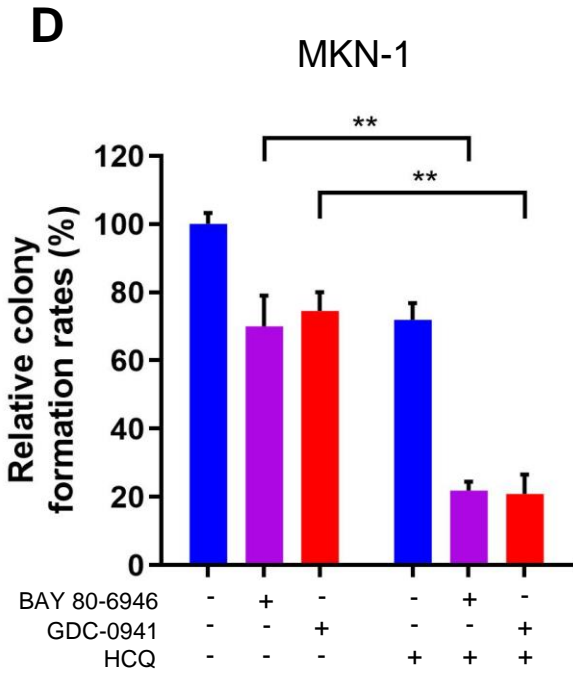

E

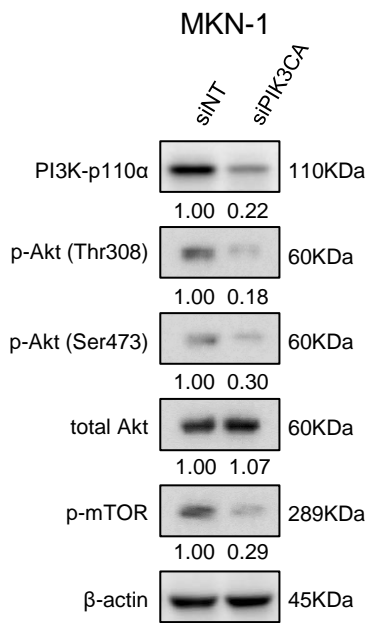

F

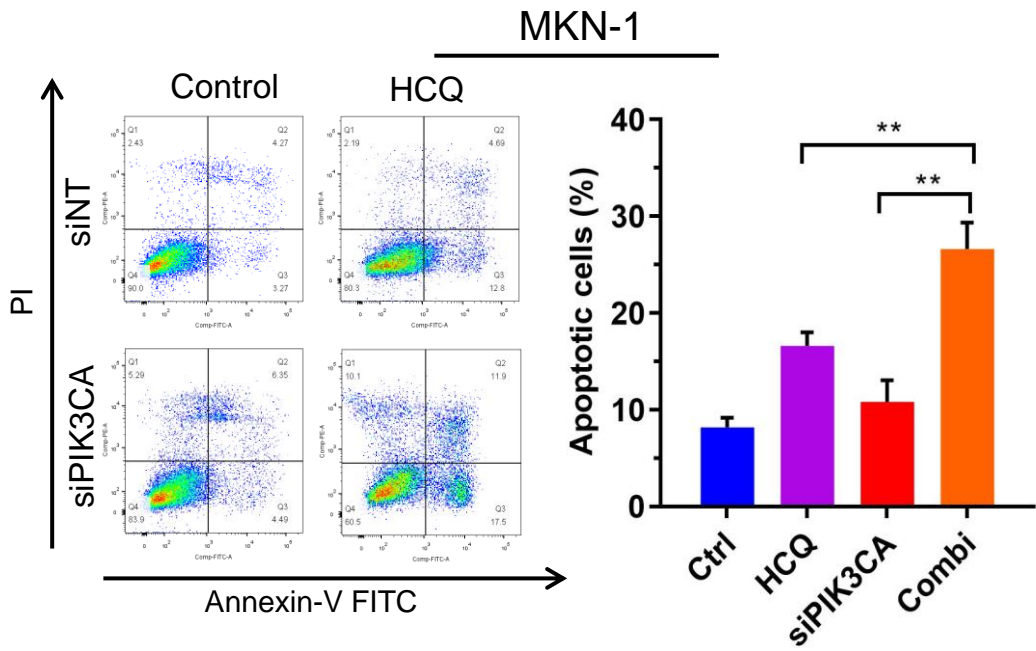

Supplement: Supplementary file 2 — Additional file 2: Figure S2. HCQ increased the sensitivity of the other PI3K inhibitors in MKN-1 cells. (A) Western blot analysis of LC3B and p62 in MKN-1 cells treated with BKM120 and/or HCQ for 48 h. (B) Cell viability was measured by PrestoBlue after treatment with BAY 80-6946/GDC-0941 alone or in combination with 20 μM HCQ for 72 h. (C) Colony-forming abilities of cells treated with HCQ and/or BAY 80-6946/GDC-0941 were determined. Colony quantification is graphed in (D). (E) Western blot analysis of PI3K-p110α, p-Akt, Akt and p-mTOR after siRNA knockdown of PI3KCA. (F) The cells were treated with HCQ for 48 h in the presence or absence of PI3KCA siRNA, and cell apoptosis was measured. Graphs are normalized to 100% per treatment and shown as mean ± SD from three independent experiments; *p < 0.05; **p < 0.01. [file 13046_2021_2176_MOESM2_ESM.pdf]

Supplementary Figure 3

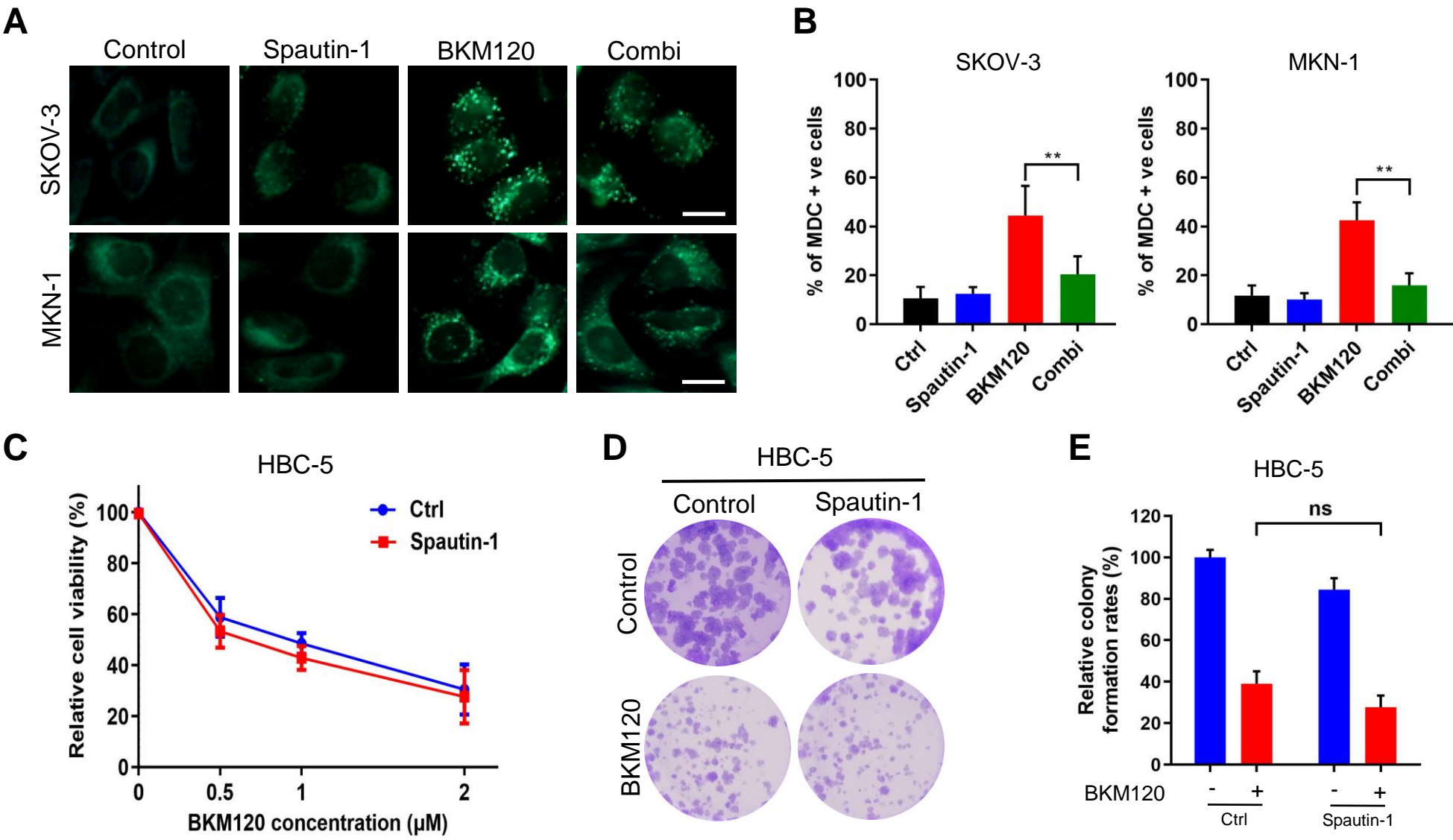

Supplement: Supplementary file 3 — Additional file 3: Figure S3. The effect of Spautin-1 on the autophagy and cytotoxity caused by BKM120. Representative images of autophagosome puncta (A) in SKOV-3 and MKN-1 treated with BKM120 (1 μM), Spautin-1 (10 μM), alone or in combination for 24 h, and the quantification (B). Scale bars, 20 μm. (C) Cell viability of HBC-5 cells after treated with different concentrations of BKM120 alone or in combination with 10 μM Spautin-1 for 72 h. (D) Colony forming ability of HBC-5 cells after treated with Spautin-1 or/and BKM120. Graph of colony quantification is shown in (E). Graphs are normalized to 100% per treatment and shown as mean ± SD from three independent experiments; ns: p > 0.05; **p < 0.01. [file 13046_2021_2176_MOESM3_ESM.pdf]

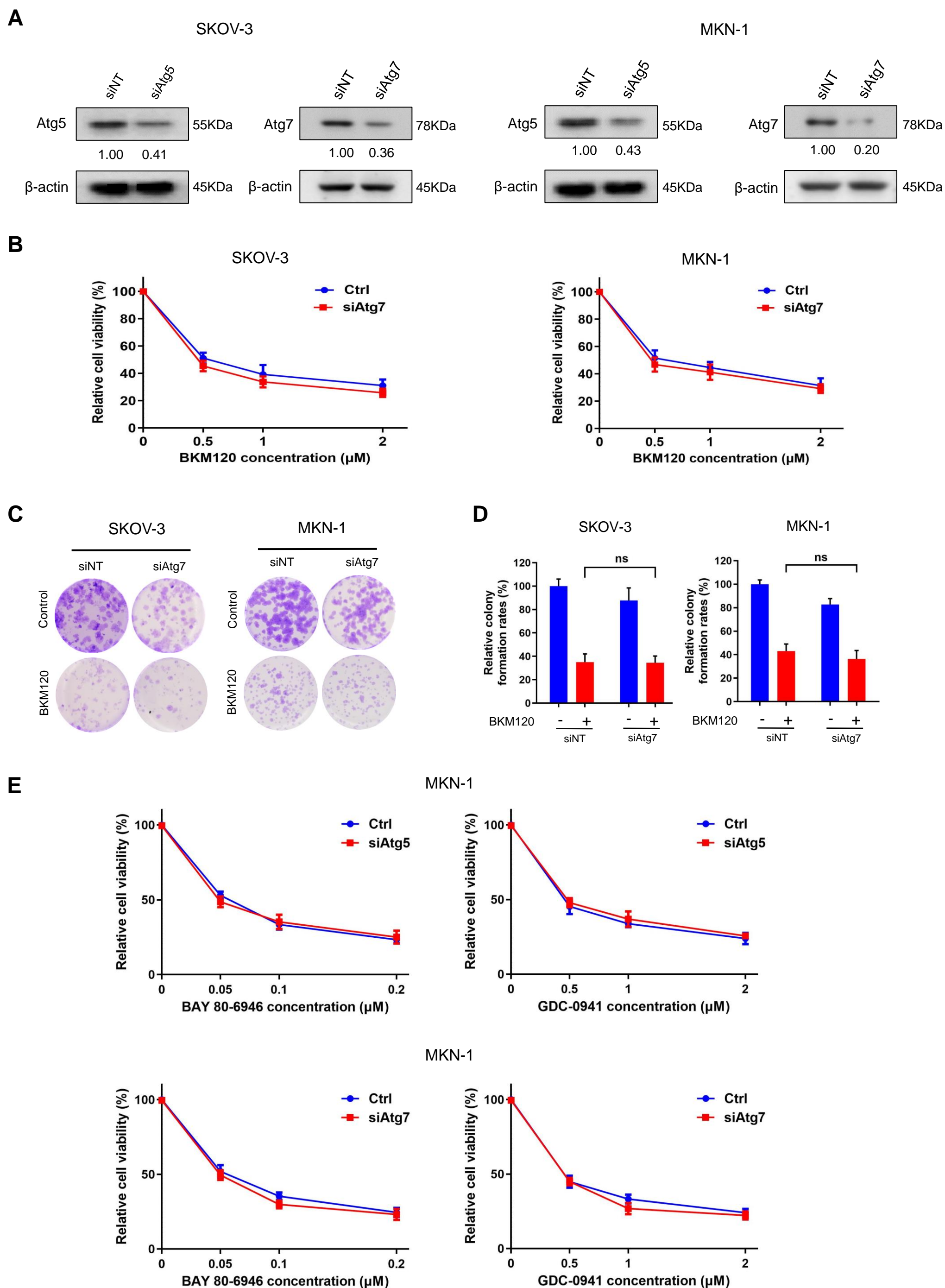

Supplement: Supplementary file 4 — Additional file 4: Figure S4. Silence of Atg5 or Atg7 did not affect the anticancer activity of BKM120 and other PI3K inhibitors. (A) Western blot analysis of Atg5 and Atg7 in SKOV-3 and MKN-1 cells after transfection with non-targeting siRNA (siNT), Atg5 and Atg7 siRNA, respectively. (B) Cell viability of SKOV-3 and MKN-1 cells after transfected with Atg7 siRNA, followed by treatment of BKM120 for 72 h. (C) Colony forming ability of SKOV-3 and MKN-1 cells after transfected with non-target siRNA or Atg7 siRNA and treated with BKM120 for 72 h and allowed to recover in fresh media for 7–10 days. Graph of colony quantification is shown in (D). (E) Cell viability of MKN-1 cells after transfected with Atg5 or Atg7 siRNA, followed by treatment of BAY 80-6946 or GDC-0941 for 72 h. Graphs are normalized to 100% per treatment and shown as mean ± SD from three independent experiments; ns: p > 0.05. [file 13046_2021_2176_MOESM4_ESM.pdf]

Supplementary Figure 5

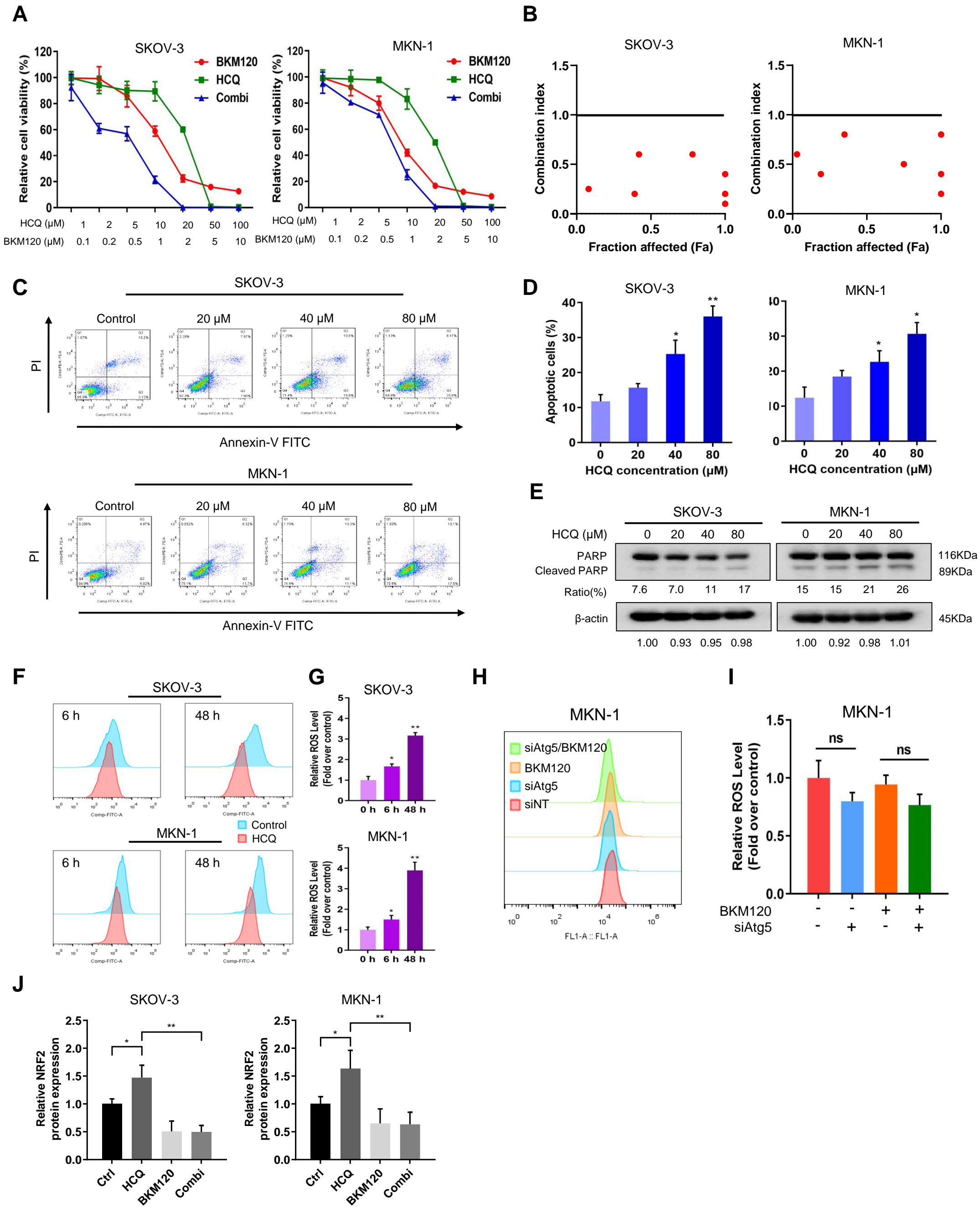

Supplement: Supplementary file 5 — Additional file 5: Figure S5. The effect of HCQ on ROS production and the synergetic effect with BKM120 on proliferation inhibition in SKOV-3 and MKN-1 cells. (A) Cell viability of SKOV-3 and MKN-1 cells after treatment with BKM120 or HCQ alone or in combination for 72 h. (B) The drug combination was analyzed using CalcuSyn software and the resulting CI-Fa plots are shown. (C) SKOV-3 and MKN-1 cells were treated with various concentrations of HCQ for 48 h and then subjected to apoptosis analysis. (D) FACS quantification of total apoptotic cell population. (E) Western blot analysis of PARP in SKOV-3 and MKN-1 cells treated with HCQ for 48 h. ROS levels (F) in SKOV-3 and MKN-1 cells were determined after treatment with 40 μM HCQ for 6 and 48 h, respectively, which were quantified as in (G). ROS level (H) in MKN-1 cells after transfected with Atg5 siRNA alone or in combination with BKM120 for 48 h was determined, and quantified as in (I). (J) Quantification of NRF2 expression after correction with the β-actin control. Graphs are shown as mean ± SD from three independent experiments; ns: p > 0.05; *p < 0.05; **p < 0.01. [file 13046_2021_2176_MOESM5_ESM.pdf]

A

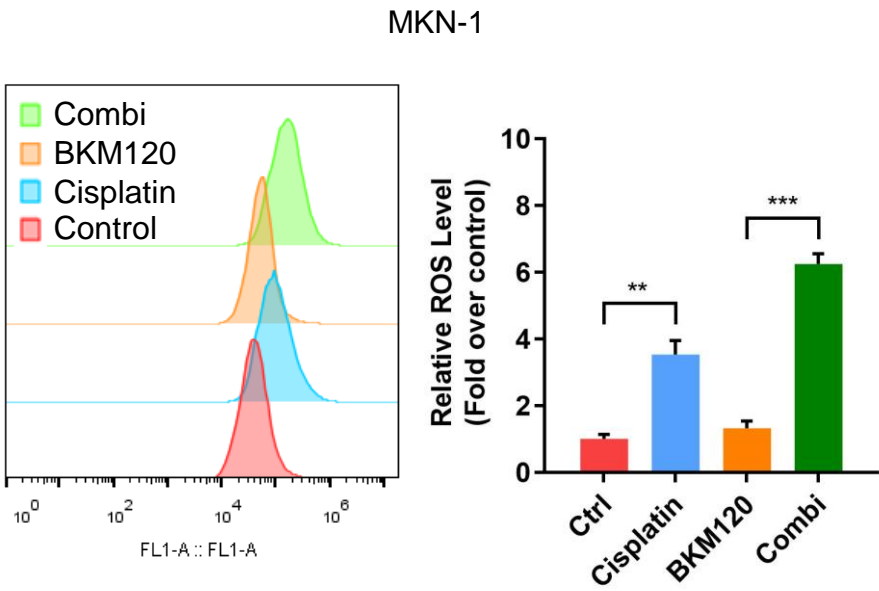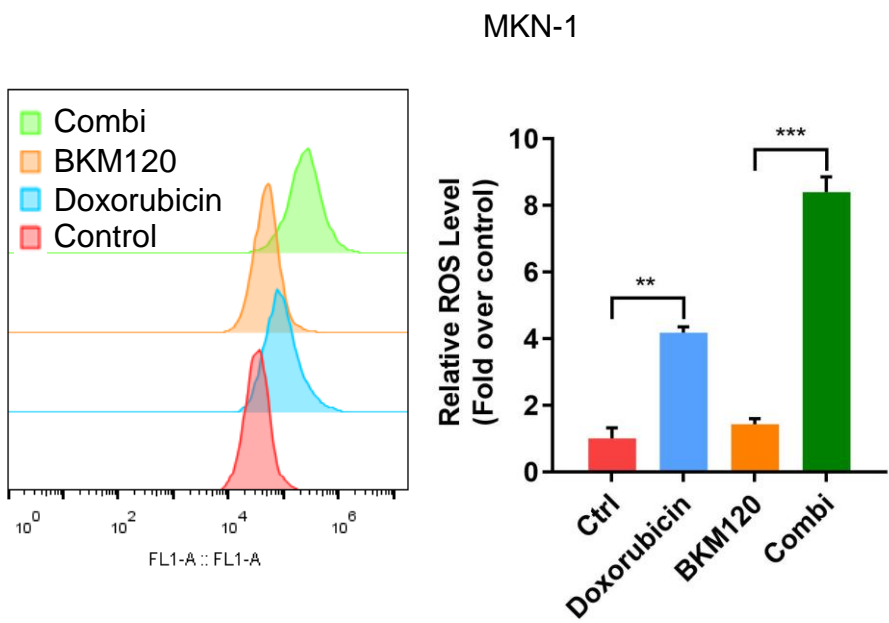

B

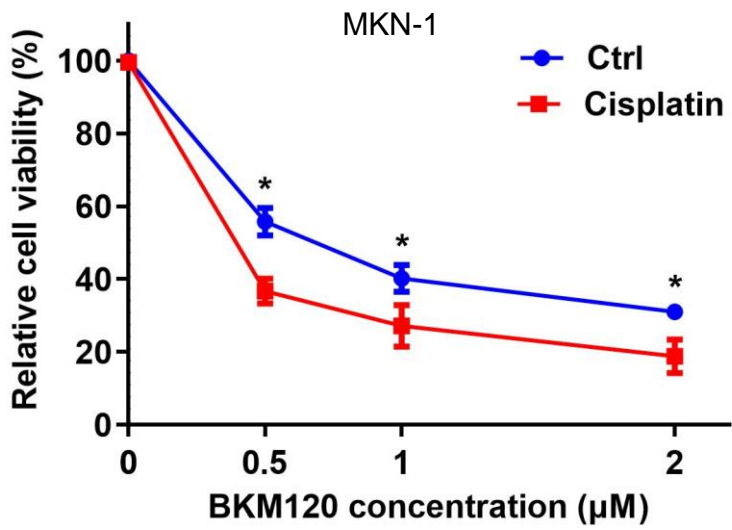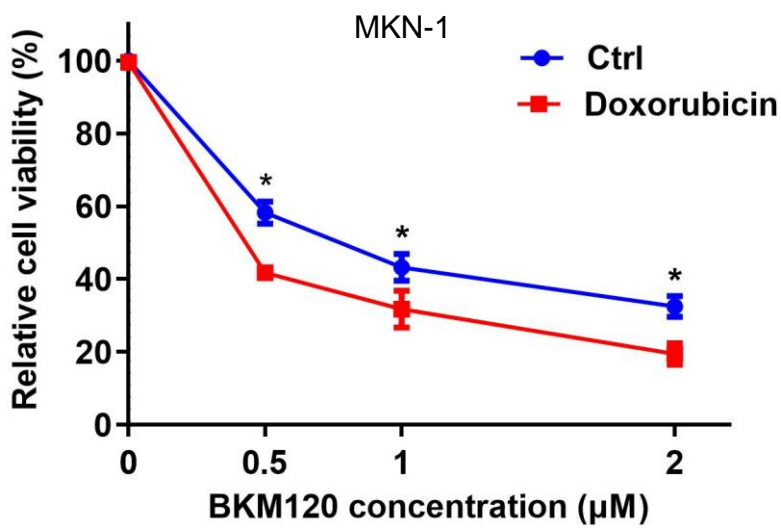

Supplement: Supplementary file 6 — Additional file 6: Figure S6. ROS inducers synergized with BKM120 to exhibit anti-proliferative effect in MKN-1 cells. (A) Determination of ROS production in MKN-1 cells treated with BKM120 and/or Cisplatin/Doxorubicin for 48 h. (B) Cell viability was measured by PrestoBlue after treatment with BKM120 alone or in combination with Cisplatin/Doxorubicin for 72 h. Graphs are normalized to the untreated group and shown as the mean ± SD from three independent experiments; *p < 0.05; **p < 0.01; ***p < 0.001. [file 13046_2021_2176_MOESM6_ESM.pdf]

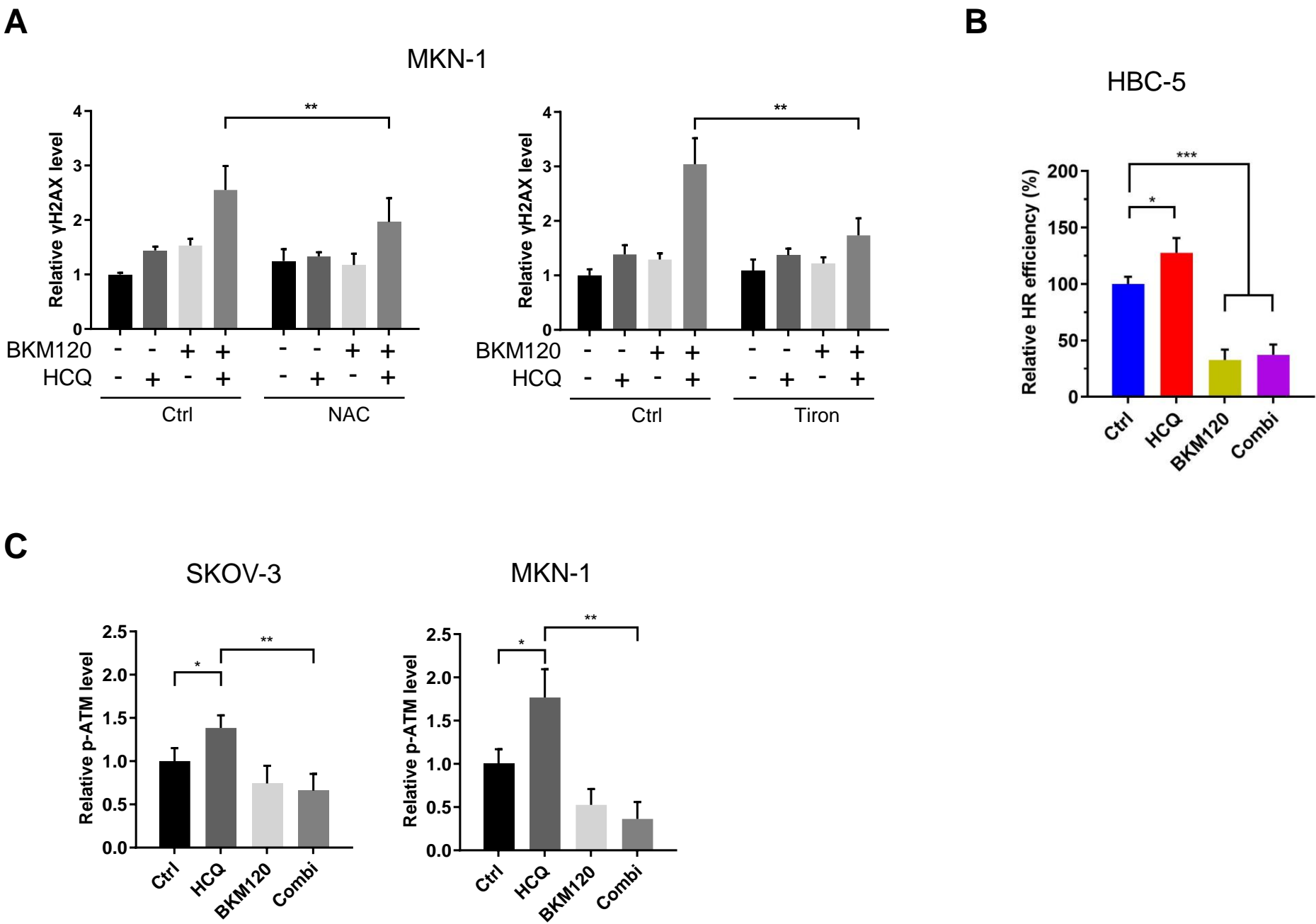

Supplement: Supplementary file 7 — Additional file 7: Figure S7. The effect of combination of HCQ and BKM120 on DSBs in tumor cells. (A) Quantification of γH2AX levels after correction with the β-actin control. (B) The HR reporter assay was used to detect the effect of the drugs on HR repair efficiency in HBC-5 cells. (C) Quantification of p-ATM levels after correction with the β-actin control; Graphs are shown as mean ± SD from three independent experiments; *p < 0.05; **p < 0.01. [file 13046_2021_2176_MOESM7_ESM.pdf]
